# Selectivity of mRNA degradation by autophagy in yeast

**DOI:** 10.1038/s41467-021-22574-6

**Published:** 2021-04-19

**Authors:** Shiho Makino, Tomoko Kawamata, Shintaro Iwasaki, Yoshinori Ohsumi

**Affiliations:** 1 Cell Biology Center, Tokyo Institute of Technology, Yokohama, Japan; 2 RNA Systems Biochemistry Laboratory, RIKEN Cluster for Pioneering Research, Saitama, Japan; 3 Department of Computational Biology and Medical Sciences, Graduate School of Frontier Sciences, The University of Tokyo, Chiba, Japan; 4 AMED-CREST, Japan Agency for Medical Research and Development, Saitama, Japan

**Keywords:** Autophagy, Ribophagy, Nutrient signalling, RNA decay

## Abstract

Synthesis and degradation of cellular constituents must be balanced to maintain cellular homeostasis, especially during adaptation to environmental stress. The role of autophagy in the degradation of proteins and organelles is well-characterized. However, autophagy-mediated RNA degradation in response to stress and the potential preference of specific RNAs to undergo autophagy-mediated degradation have not been examined. In this study, we demonstrate selective mRNA degradation by rapamycin-induced autophagy in yeast. Profiling of mRNAs from the vacuole reveals that subsets of mRNAs, such as those encoding amino acid biosynthesis and ribosomal proteins, are preferentially delivered to the vacuole by autophagy for degradation. We also reveal that autophagy-mediated mRNA degradation is tightly coupled with translation by ribosomes. Genome-wide ribosome profiling suggested a high correspondence between ribosome association and targeting to the vacuole. We propose that autophagy-mediated mRNA degradation is a unique and previously-unappreciated function of autophagy that affords post-transcriptional gene regulation.

## Abstract (web summary)

Autophagic degradation of proteins and organelles is well studied, but the role of autophagy in RNA regulation is unclear. Here, the authors profile mRNAs targeted to the vacuole in yeast and observe autophagy-mediated mRNA degradation is not random but rather is a preferential process.

## Introduction

Autophagy is a highly conserved eukaryotic pathway that isolates cellular components for degradation and recycling in response to nutrient starvation, thereby maintaining homeostasis in nutrient-limited environments^1,2^. Upon autophagy induction, substrates are sequestered within a double-membrane vesicle called an autophagosome and subsequently delivered to the vacuole (yeast) or lysosome (mammals) where they are degraded by hydrolytic enzymes.

Autophagy is originally thought to be nonselective (i.e., bulk), which isolates cytoplasmic material in an apparently random manner. In contrast, selective autophagy eliminates harmful proteins and superfluous, damaged organelles, such as endoplasmic reticula (ER) and mitochondria^3^. Substrates of selective autophagy, often marked for degradation by modifications such as ubiquitination, are recognized through their binding to receptors that facilitate localization to the site of autophagosome formation^4–6^. In addition to the clear distinction of substrate, autophagy may show the broad spectrum of protein substrate preference. Acetaldehyde dehydrogenase and tRNA ligases^7,8^ are such examples. The mechanism underlying this preference has been still unknown.

Despite the many studies of protein and organelle degradation by autophagy, RNA degradation by autophagy has not been well examined. Historically, RNA degradation by autophagy was suggested in pioneering works examining amino acid-starved rat livers by Lardeux and Mortimore^9–12^. More recently, our group showed that RNA delivered to vacuoles via autophagy is degraded by the T2-type RNase Rny1, and that the resulting nucleotides are further hydrolyzed to nucleosides by the vacuolar phosphatase/nucletidase Pho8^13^. The question of whether RNA degradation by autophagy occurs preferentially, however, remains unaddressed.

In this study, we examine the degradation of mRNAs by rapamycin-induced autophagy in yeast. mRNA sequencing reveals that autophagy preferentially delivers a subset of mRNA species to the vacuole for Rny1-mediated degradation. We further find that the selectivity of autophagy-mediated mRNA degradation is coupled with translation by reporter gene analysis. Genome-wide ribosome profiling further demonstrates that persistent ribosomal interaction is associated with selective mRNA delivery to vacuoles. The autophagic delivery of ribosome-mRNA complexes to the vacuole depends on Atg24 sorting nexin complex, which is required for autophagic degradation of multisubunit complexes, such as the proteasome and ribosome. We provide a foundational description of autophagy-mediated RNA degradation in yeast, as well as molecular insights into the selectivity of this hitherto underappreciated pathway.

## Results

### Autophagy delivers mRNAs to the vacuole for degradation

**Fig. 1:**
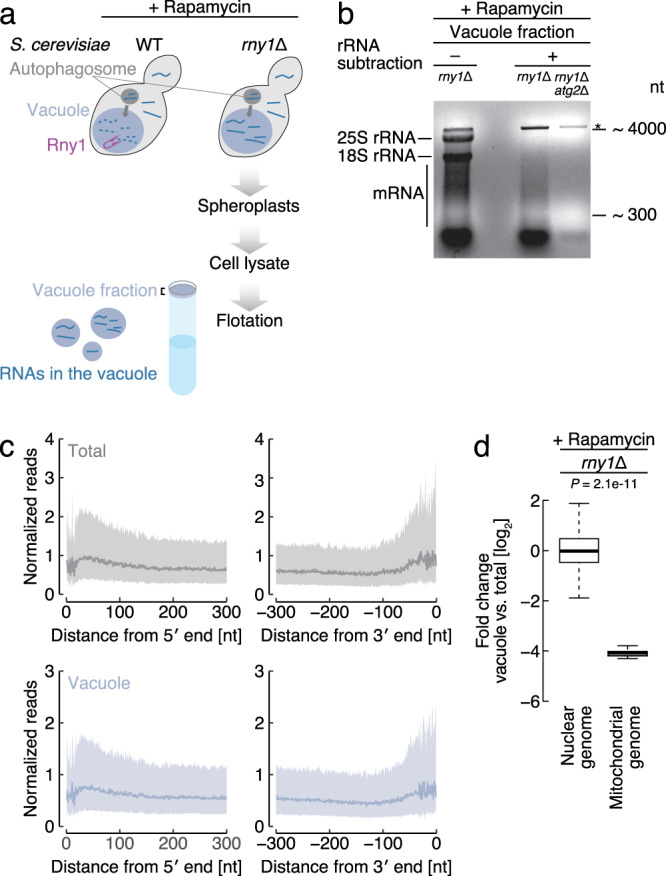
Determination of the autophagy-dependent vacuolar transcriptome. **a** Schematic illustration of the experimental design. Following the induction of autophagy by 3 h rapamycin treatment, vacuoles from yeasts were purified by ultracentrifugal flotation. A strain deleted for the vacuolar nuclease Rny1, which degrades nucleic acids within the vacuole, was used. **b** Autophagy-dependent mRNA accumulation in the vacuole fraction. Agarose gel electrophoresis image of RNAs detected in the vacuole fraction of rapamycin-treated (3 h) cells. rRNA subtraction was performed by treating extracted RNA samples with oligonucleotides complementary to rRNAs. Representative results from three independent experiments are shown. Asterisk indicates a nonspecific band. **c** Meta-gene analysis for mRNA-Seq read distributions around the 5′ and 3′ ends of transcripts from whole-cell lysate (gray) or vacuole fraction (purple). The 5′ end of reads is depicted. The line and shaded area represent the median and interquartile range, respectively. **d** Change in mitochondrial genome-encoded mRNAs’ enrichment in vacuoles versus total cellular extracts following 3 h rapamycin treatment. Box plots show the accumulation of reads originating from nuclear and mitochondrial genomes, as determined by RNA-Seq. The median, interquartile range (IQR), and 1.5 IQR are represented by a solid line, box, and whiskers, respectively. Significance was determined by unpaired two-sided Mann-Whitney U-test.

We began by identifying mRNAs species delivered to vacuoles by autophagy. As mRNA delivered to the vacuole is immediately degraded by the vacuolar ribonuclease Rny1 in wild-type (WT) cells^13^, we constructed a strain (*rny1*Δ) lacking this enzyme. To isolate vacuole-delivered mRNAs, we adopted a strategy employing a highly purified vacuolar fraction from cell lysates by flotation with ultracentrifugation (Fig. 1a). The purity of the vacuolar fraction was confirmed by vacuole protein enrichment and the apparent absence of ER, Golgi, and nucleolar proteins (Supplementary Fig. 1a).

Using rapamycin to induce autophagy through TORC1 suppression^14^ and the vacuole purification method described above, we examined the accumulation of RNA species in vacuoles. In addition to rRNAs, we also detected mRNAs that were clearly observed following rRNA subtraction (Fig. 1b). Importantly, we determined that the presence of mRNAs in vacuoles depends on autophagy: mRNAs were hardly detected in *atg2*Δ cells, which are completely defective for autophagy^15^.

Next, we characterized the mRNAs in the vacuoles by RNA sequencing. The even distribution of vacuolar mRNA reads suggests that nontruncated, full length mRNAs are delivered to vacuoles in *rny1*Δ cells (Fig. 1c) and verifies that Rny1 is the sole vacuolar nuclease responsible for mRNA degradation^13^. In contrast to nuclear genome-encoded mRNAs, mitochondrial genome-encoded mRNAs were clearly excluded from the vacuole fraction (Fig. 1d), suggesting only limited mitochondrial degradation under the employed conditions.

### Vacuolar delivery of mRNA has selectivity

**Fig. 2:**
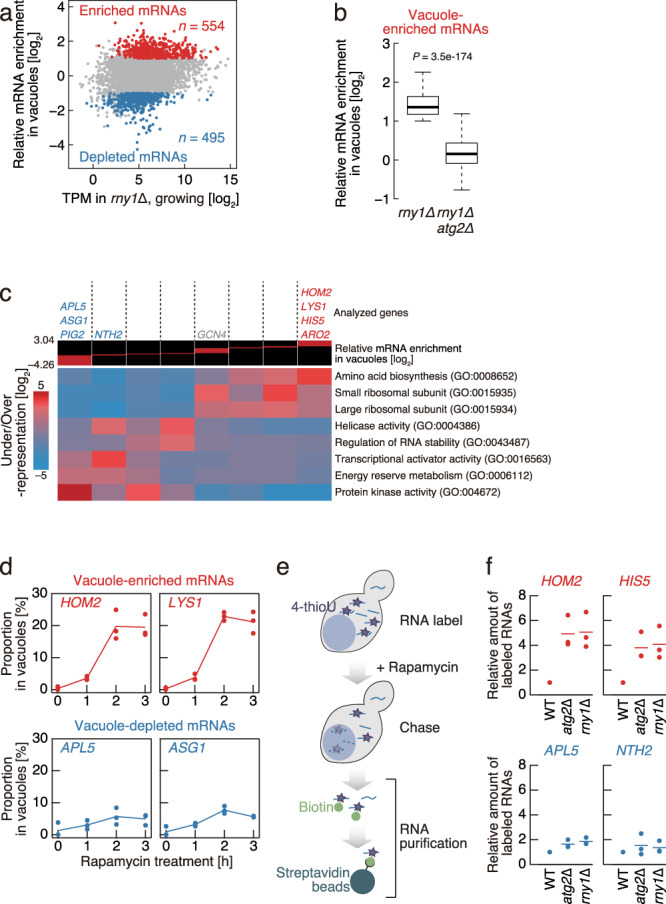
Autophagic mRNA delivery into vacuoles has selectivity. **a** Relative rapamycin-induced mRNA delivery into vacuoles. MA (log ratio vs. mean average) plot of 5592 mRNAs showing relative mRNA enrichment in vacuoles following rapamycin treatment (3 h) versus transcript per million (TPM) in *rny1*Δ cells in growing condition. mRNAs identified as deviating significantly from total cell extracts were classified as “enriched” (log_2_-fold change ≥ 1 and *q* value < 0.01) or “depleted” (log_2_-fold change ≤ −1 and *q* value < 0.01), as indicated in red and blue, respectively. **b** Autophagy dependency of vacuole-enriched mRNA delivery to the vacuole. Box plot showing the relative mRNA enrichment of vacuole-enriched mRNAs following rapamycin treatment (3 h) in *rny1*Δ and *rny1*Δ *atg2*Δ cells. The median, IQR, and 1.5 IQR are represented by solid line, box, and whiskers, respectively. Significance was determined by unpaired two-sided Mann-Whitney U-test. **c** Gene ontology analysis of mRNAs by relative vacuolar enrichment, as determined by iPAGE^62^. The representative vacuole-enriched (red) or -depleted (blue) genes analyzed in this study are shown. **d** Time course of vacuole-enriched (red) or -depleted (blue) mRNA accumulation in *rny1*Δ cell vacuoles following rapamycin treatment. **e** Overview of a labeling strategy employing 4-thiouracil (4-thioU) with rapamycin treatment to monitor mRNA degradation. **f** Persistence of 4-thioU-labeled vacuole-enriched (red) or -depleted (blue) mRNAs following 1 h rapamycin treatment as determined by the strategy shown in **e**. Data were normalized to 4-thioU-labeled mRNA values detected in WT cells, which were set to 1. In **d** and **f**, data present mean (line) and individual results (points) of three independent experiments.

We next set out to determine unique features of mRNA species delivered to the vacuole while accounting for previously reported dramatic changes in the cellular transcriptome upon rapamycin treatment (Supplementary Fig. 2a and see Materials and Methods)^16^. A broad spectrum of mRNAs is delivered to the vacuole (Fig. 2a and Supplementary Fig. 2b). We identified quantitatively over- and under-represented subsets of mRNAs relative to the cellular transcriptome, which we refer to as vacuole enriched and vacuole depleted, respectively. Notably, this analysis further ensured that the delivery of vacuole-enriched mRNAs is dependent on Atg2 (Fig. 2b). Northern blotting of representative vacuole-enriched mRNAs further confirmed autophagy-dependent mRNA enrichment in the vacuole (Supplementary Fig. 2c).

Next, we conducted functional characterization of the vacuole-enriched and -depleted mRNAs by gene ontology analysis. Strikingly, housekeeping mRNAs associated with amino acid biosynthesis and ribosomal proteins were most likely to be delivered to vacuoles (Fig. 2c). In contrast, the vacuole-depleted fraction was characterized by mRNAs with regulatory roles, such as those encoding proteins with protein kinase, energy reserve metabolism, and transcriptional activator (Fig. 2c). We note that the mRNA selectivity is independent of basal mRNA abundance in cells (Fig. 2a). Taken together, these results reveal that autophagy-mediated mRNA delivery to vacuoles is selective, not random, in nature.

We quantified the proportion of mRNA delivered to the vacuole. To this end, we used the activity of vacuolar alkaline phosphatase^17,18^ as a standard to ascertain the recovery of vacuoles from cell lysates and thereby the relative quantity of mRNAs delivered to the vacuole. This strategy revealed that for vacuole-enriched mRNA species, the proportion of mRNAs accumulating in the vacuole increased dramatically upon autophagy induction (Fig. 2d and Supplementary Fig. 2c); more than 20% of vacuole-enriched *HOM2* (aspartic beta semi-aldehyde dehydrogenase) and *LYS1* (saccharopine dehydrogenase) mRNAs in cell lysates were delivered to vacuoles following 3 h of rapamycin treatment. In contrast, the fraction of vacuole-depleted species, such as *APL5* (a subunit of the clathrin associated protein complex) and *ASG1* (zinc cluster protein proposed to be a transcriptional regulator), was limited at most to a 5% increase in vacuolar localization (Fig. 2d and Supplementary Fig. 2d).

As vacuole-enriched mRNAs are preferentially delivered to the vacuole, we reasoned that the relatively high rate of degradation of these mRNAs should be observed in WT cells. This was verified by RNA-Seq analyses of WT total cell lysates: the total abundance of vacuole-enriched mRNAs was significantly decreased following rapamycin treatment (Supplementary Fig. 2e, f). To more directly monitor mRNA degradation, we labeled cellular mRNAs with 4-thiouracil (4-thioU) in vivo, after which we subjected cells to chase in uracil-containing media (Fig. 2e). For this experiment, we used *ura3*Δ cells to enhance incorporation of modified uracil into mRNAs (Supplementary Fig. 3a). In addition, we ascertained that the deletion of *ATG2* or *RNY1* does not affect the RNA labeling rate and total mRNA levels with 4-thioU (Supplementary Fig. 3a, b). Following 1 h rapamycin treatment, labeled mRNAs were biotinylated and purified using streptavidin beads, followed by quantification of mRNA abundance. As the reliability of short-lived mRNA analysis is poor, we focused on relatively long-lived mRNAs^19^. Two representative vacuole-enriched mRNAs engaged in amino acid biosynthesis, *HOM2* and *HIS5* (histidinol-phosphate aminotransferase), were detected at between 3- to 4-fold abundance in *atg2*Δ cells in comparison to WT cells (Fig. 2f, top), providing further evidence of autophagy-dependent degradation. Remarkably, even with an intact autophagy machinery, vacuole-enriched mRNAs in *rny1*Δ cells were also retained to a nearly identical degree as that observed in *atg2*Δ cells, indicating that the nuclease activity of Rny1 is responsible for the degradation of mRNAs following delivery to the vacuole. Meanwhile, vacuole-depleted *APL5* and *NTH2* (putative neutral trehalase) mRNAs showed only marginal autophagy-dependent differences in stability (Fig. 2f, bottom). These results highlight the impact of autophagy on mRNA degradation and clearly demonstrate that autophagic mRNA degradation is carried out via preferential vacuole delivery and subsequent Rny1-mediated hydrolysis.

### Preferential mRNA delivery to vacuoles is coupled with translation

As for other forms of selective autophagy in protein degradation, the mRNA degradation pathway must employ a distinct mechanism. By comparing vacuolar mRNA delivery and mRNA stability, we did not find the correspondence between selectivity of mRNA delivery to the vacuole and the steady-state half-life of mRNAs^19^ (Supplementary Fig. 4a). Thus, the recognition and isolation of target mRNAs necessary for vacuolar degradation appeared to be different to canonical cytoplasmic mRNA turnover, leading us to investigate the mechanistic basis of vacuolar mRNA degradation.

Selective degradation of the ER has been reported^20,21^, and many mRNAs are bound to the ER surface^22^. We determined that concomitant degradation of ER-bound mRNAs is unlikely as ER-bound mRNAs were not enriched in vacuoles (Supplementary Fig. 4b). Another possibility is that mRNAs within ribonucleoprotein (RNP) granules, such as stress granules, are degraded by autophagy through their association with RNP granules^23,24^. However, stress granule mRNAs^25^ were not enriched in the vacuole; rather, these mRNAs were predominantly identified in the vacuole-depleted fraction, suggesting that stress granules are not degraded by autophagy under the conditions employed in this study (Supplementary Fig. 4c).

**Fig. 3:**
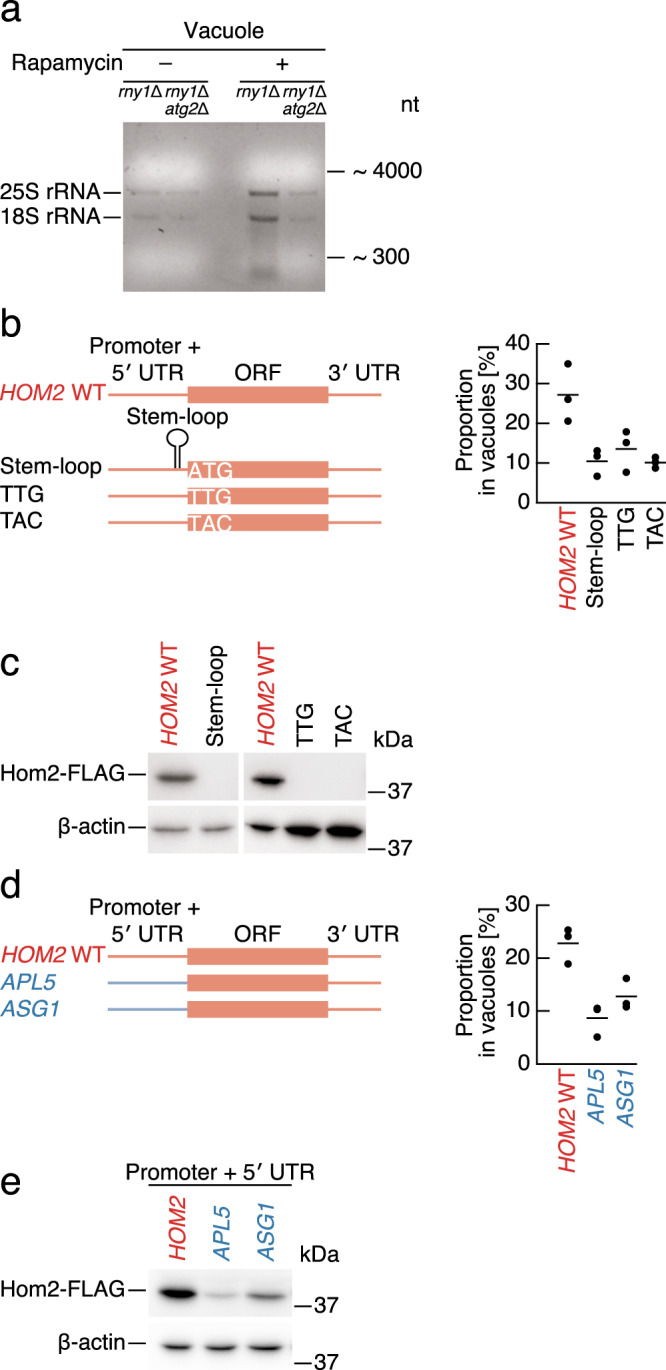
Autophagy-mediated mRNA degradation is coupled with translation. **a** Autophagy-dependent rRNA accumulation in the purified vacuole fraction. Agarose gel electrophoresis of RNAs from purified vacuoles with or without 3 h rapamycin treatment. Representative results from three independent experiments are shown. **b, d** Proportion of a representative vacuole-enriched reporter mRNA*, HOM2*, recovered from vacuolar fractions following 3 h rapamycin treatment. Data were analyzed as described in Fig. 2d. In **b**, translation was blocked either through the introduction of an inhibitory stem-loop just before the *HOM2* start codon, or by introducing point mutations into the start codon (ATG to TTG or TAC). In **d**, the promoter-5′ UTR region of *HOM2* was replaced with that of vacuole-depleted *APL5* or *ASG1*. Data present mean (line) and individual results (points) of three independent experiments. **c, e** Protein levels of reporter mRNAs used in **b** and **d** as determined by Western blotting in growing condition. Representative results from two independent experiments are shown.

Autophagy is also known to sequester cytoplasmic ribosomes into autophagosomes for degradation in the vacuole^26^. We observed the accumulation of rRNA in the vacuolar fraction following autophagy induction (Fig. 3a), giving rise to the possibility that translating mRNAs are delivered to the vacuole. To test this hypothesis, we designed a reporter assay to monitor mRNA enrichment by using the vacuolar delivery of *HOM2* mRNA as a model (Fig. 2c, d, and Supplementary Fig. 2c, d). We generated and expressed a series of reporter plasmids under the control of the native *HOM2* promoter (ensuring endogenous expression levels) in a *hom2*Δ background strain (Fig. 3b). Expression of WT *HOM2* mRNA in this strain recapitulated vacuolar delivery. In contrast, delivery of reporter mRNA to the vacuole was inhibited by the insertion of a strong stem-loop just before the start codon, which blocks start codon scanning by the pre-initiation 43 S complex and thus impedes translation^27,28^ (Fig. 3b, c). Further, blocking of translation by the elimination of the start codon (substitution to TTG or TAC) also reduced mRNA delivery into vacuoles while inhibiting translation (Fig. 3b, c). Reporter mRNA level in total cell lysate were comparable with WT *HOM2* mRNA (Supplementary Fig. 5d). Alternative vacuole-enriched *ARO2* (chorismite synthase/flavin reductase) mRNA, which was similarly engineered as *HOM2* reporter, also showed translation dependency for vacuolar delivery (Supplementary Fig. 5a, b, e). These results suggest that mRNA delivery to the vacuole is closely coupled with translation.

To determine whether a protein-encoding region of vacuole-enriched mRNAs is responsible for autophagic delivery to the vacuole, we swapped the *HOM2* ORF with an ORF encoding GFP. Substitution of the *HOM2* ORF had little effect on vacuolar delivery (Supplementary Fig. 5c). In addition, the substitution of the 3′ UTR of *HOM2* with that of *PIG2* (putative type-1 protein phosphatase targeting subunit), a representative vacuole-depleted mRNA, had only a very weak effect on vacuolar delivery (Supplementary Fig. 5c, f). We next swapped the *HOM2* 5′ UTR with vacuole-depleted mRNA 5′ UTRs. We employed 5′ UTRs from vacuole-depleted mRNAs expressed at a similar mRNA level to WT *HOM2* mRNA, since a low abundance of reporter mRNAs could result in the overestimation of vacuolar delivery (Supplementary Fig. 5g). The substitution of the *HOM2* 5′ UTR with vacuole-depleted *APL5* or *ASG1* 5′ UTRs clearly reduced the efficiency of delivery (Fig. 3d). Importantly, the 5′ UTR of vacuole-depleted mRNAs was associated with a marked decrease in *HOM2* protein level (Fig. 3e), which is consistent with the coupling of translation and degradation. Thus, we propose that the 5′ UTR is critical for vacuolar delivery through its regulation of vacuole-enriched mRNA translation.

### Ribosome-mRNA association enhances vacuolar delivery by autophagy

**Fig. 4:**
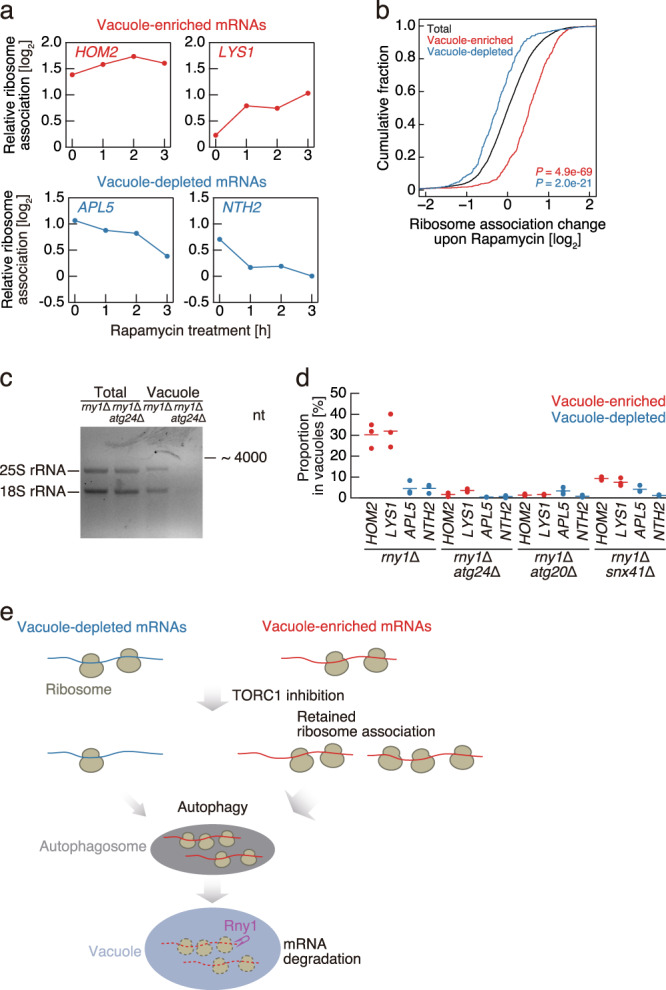
Ribosomal association enhances mRNA delivery to the vacuole. **a** Ribosome association (ribosome footprint data normalized to RNA-Seq reads) of the vacuole-enriched (red) or -depleted (blue) mRNAs (defined in Fig. 2a) during rapamycin treatment. **b** Cumulative distribution of vacuole-enriched (red) and -depleted mRNAs (blue) (defined in Fig. 2a) in relation to the change in ribosome association following 3 h of rapamycin treatment. Significance was determined by unpaired two-sided Mann-Whitney U-test. **c** Agarose gel electrophoresis of RNAs recovered from whole-cell lysate or purified vacuoles of *rny1*Δ and *rny1*Δ *atg24*Δ cells following 3 h rapamycin treatment. Representative result from three independent experiments is shown. **d** The vacuole-enriched (red) or -depleted (blue) mRNA accumulation in purified vacuoles of *rny1*Δ, *rny1*Δ *atg24*Δ, *rny1*Δ *atg20*Δ, and *rny1*Δ *snx41*Δ cells. Data were analyzed as described in Fig. 2d. Data present mean (line) and individual results (points) of three independent experiments. **e** A model for autophagy-mediated mRNA degradation in yeast. A subset of mRNAs is preferentially delivered to vacuoles by autophagy. This mRNA delivery is coupled to mRNA translation. Persistence of ribosome association with mRNA enhances selective mRNA delivery to vacuoles even during blockage of global protein synthesis by TORC1 inhibition. mRNAs delivered to the vacuole by autophagy are subsequently degraded by the nuclease Rny1.

We next conducted an analysis of ribosome-mRNA interactions at the genome-wide level. To this end, we performed ribosome profiling^29^ and monitored associations upon rapamycin treatment, normalizing data to mRNA abundances obtained by RNA-Seq. Rapamycin treatment reduced global protein synthesis, as indicated by reduced polysomes formation (Supplementary Fig. 6a) and previously reported^30^. Remarkably, ribosomal association with vacuole-enriched *HOM2* and *LYS1* mRNAs persisted during rapamycin treatment (Fig. 4a). In contrast, vacuole-depleted *NTH2* and *APL5* mRNAs exhibited a time-dependent decrease in ribosomal association (Fig. 4a). These trends were observed with a high degree of reproducibility throughout all vacuole-enriched and -depleted transcripts (Fig. 4b) and at all assessed time points following rapamycin treatment (1, 2, and 3 h) (Supplementary Fig. 6b). Taken together, these results strongly suggest that persistent ribosome-mRNA association is a key determinant of mRNA degradation by autophagy, even as global translation activity is inhibited during rapamycin treatment.

A previous study reported that Atg24 (also known as Snx4) is specifically required for the autophagic degradation of multisubunit complexes such as ribosomes, but not of bulk proteins^31^. Given that autophagic degradation of mRNA is coupled to ribosomal association, we hypothesized that Atg24 is required for autophagy-mediated RNA degradation. To test this, we assessed autophagic degradation of GFP-fused proteins by western blotting, whereby autophagic delivery to the vacuole and subsequent degradation of a protein is detected by the liberation of the vacuolar protease-resistant GFP moiety. As reported previously^31^, the cleavage of free GFP from the bulk-autophagy reporter Pgk1-GFP was observed in both *atg24*Δ and WT cells (Supplementary Fig. 7a), whereas cleavage of GFP from the ribosomal protein Rpl37a-GFP was detected in WT but not *atg24*Δ cells (Supplementary Fig. 7b). Critically, accumulation of rRNAs in the vacuoles of *rny1*Δ*atg24*Δ double mutant cells was not observed (Fig. 4c), indicating that ribosomal degradation by autophagy is largely dependent on Atg24. Quantification of the amount of mRNA delivered to the vacuole suggests that vacuolar mRNA delivery was severely inhibited in the absence of Atg24 following rapamycin treatment (Fig. 4d).

Atg24 consists of a sorting nexin complex with Atg20 or Snx41^32–34^. We also tested the impact of Atg20 and Snx41 for vacuolar delivery of mRNAs. Clearly, the deletion of these genes reduced the efficiency of mRNA delivery to the vacuole (Fig. 4d), suggesting that Atg24 functions as complexes with Atg20 or Snx41 for autophagy-mediated mRNA delivery into vacuoles. Overall, we conclude that ribosome-bound mRNAs are preferentially delivered to vacuoles by the autophagy machinery and facilitate their degradation (Fig. 4e).

## Discussion

Whereas cytoplasmic RNA degradation pathways, including canonical mRNA decay and the mRNA surveillance system, have been well studied, vacuolar degradation of mRNAs mediated by autophagy remains poorly understood. In this study, we demonstrate the delivery of mRNAs to vacuoles by autophagy, and that this process is mRNA-selective in nature (Fig. 4e). Following TOR inhibition, a subset of mRNAs, including those implicated in amino acid biosynthesis and ribosomal protein-encoding mRNAs, are delivered by rapamycin-induced autophagy to the vacuole, where they are degraded by the vacuolar nuclease Rny1. The delivery of mRNAs requires Atg24 and is enhanced by their ribosomal association, the latter of which is mediated by the 5′ UTR. The negative correlation between vacuole delivery and stress granule enrichment (Supplementary Fig. 4c) may reflect the relatively low translation rate of mRNAs within stress granules, thereby likely inhibiting autophagy-mediated vacuolar delivery.

Considering that mRNAs are apparently delivered to the vacuole in the form of a polysome, the selective degradation of ribosomes by autophagy (ribophagy)^35^ offers one potential explanation for the degradation of mRNAs observed in this study. Previous works reported that ribophagy occurs following a long period (~24 h) of nitrogen starvation^35–38^. However, in this study we observe the delivery of mRNAs to vacuoles at much earlier time points (at 3 h) by chemical TORC1 inhibition (Fig. 2)^13^. Moreover, while ribophagy has been reported to depend on the Ubp3/Bre5 deubiquitination complex^35^, RNAs are still delivered into vacuoles even in the absence of these factors^13^. Although the relation between translation status and ribophagy has not been well studied, the preferential mRNA degradation that we found in this study cannot be attributed to previously described ribophagy^35^. While the clear sequestration of ribosomes within vacuoles has been reported^26^, whether mammalian ribophagy occurs is currently a point of contention. While one study reported containing ribosomes within autophagosomes and attributed their sequestration into autophagosomes to the receptor protein NUFIP1^39^, another showed only limited degradation of ribosomes by autophagy during nutrient stress^40^. Meanwhile, the proportion of ribosomal protein in yeast is an order of magnitude higher than that of mammalian cells^41^. Ribophagy may therefore differently contribute to ribosomal turnover and mRNA degradation in various species.

There are two potential explanations for persisted ribosome-mRNA association during rapamycin treatment: increased translation initiation or reduced translation elongation, with the former being the most straightforward interpretation of the data presented in this study. In the latter case, reduced translation elongation would result in a high representation of ribosome footprints proximal to the 5′ end of ORFs^42^. We assessed the position of ribosome association on mRNAs by calculating polarity score^42^, but did not observed distribution of ribosome footprints proximal to the 5′ end of ORFs in vacuole-enriched mRNAs (Supplementary Fig. 8a). Enhanced translation initiation is also consistent with the important role of 5′ UTRs in translation: these regions possess important regulatory elements that determine the rate of translation initiation^43^. The upstream ORF (uORF), a region found within the 5′ UTR of genes such as *GCN4* (transcriptional activator of amino acid biosynthetic genes)^44,45^, allows preferential translation under stress^46^ and may support such enhanced translation initiation (Supplementary Fig. 8b, c). However, ribosome profiling did not identify any uORFs among vacuole-enriched mRNAs (Supplementary Fig. 8d). We only observed that small differences in the length of 5′ UTR^47^ between vacuole-enriched and vacuole-depleted mRNAs (Supplementary Fig. 9a, b). RNA binding protein(s) that interact with the 5′ UTR of implicated mRNAs may also facilitate recognition by the autophagy machinery, although we did not find any consensus sequences by motif analysis of vacuole-enriched mRNAs.

Another question is how delivered translating ribosomes or ribosome-mRNA complexes (polysomes) are preferentially recognized by the autophagy machinery. Interaction with adaptor proteins, as observed in selective autophagy^4–6^, or specific, transient protein/RNA modifications^48,49^ may act as intermediaries that recruit the autophagy machinery for mRNA degradation. In any case, our data imply that the state of polysomes in the cytoplasm is not homogeneous and dynamically changes under cellular conditions.

In this study, we induced autophagy using rapamycin, a pharmacological inhibitor of TORC1 widely used in autophagy research, but the investigation of mRNA degradation selectivity under other conditions promises to shed further light on interplay between ribosomal association and mRNA delivery. Using this strategy to understand the global landscape of vacuole-enriched mRNAs under a range of autophagy-inducing conditions may provide a generalized rationale for mRNA preference.

We propose that autophagy acts as an mRNA degradation system at the translation step, adding a mechanism for transcriptional regulation and global translation inhibition during cellular adaptation to stress conditions. The autophagic degradation of ribosome-associated mRNAs in the early phase of the stress response may be required to facilitate the shift to translation of stress response genes. Degradation of mRNAs engaged in ribosome association may be counter-intuitive, as this may suggest inefficient adaptation to stress when the cell is least able to tolerate the waste of resources. However, such degradation likely reflects an intricate balance between the expression of target genes and the inhibition of needless expression of target mRNAs. This study demonstrates that autophagy is involved in the regulation of gene expression through mRNA degradation, thereby playing a critical role in cellular homeostasis.

## Methods

### Yeast strains and media

Strains used in this study are listed in Supplementary Table 1. Gene disruption and tagging were performed using a standard PCR-based method^50,51^. Cells were grown in rich medium (1% yeast extract, 2% peptone, and 2% glucose) or synthetic defined casamino acid medium without uracil (SDCA-uracil: 0.17% Difco yeast nitrogen base without amino acids and ammonium sulfate, 0.5% casamino acids, 0.5% ammonium sulfate, 0.002% tryptophan, 0.002% adenine, and 2% glucose). Yeast cells were grown in liquid media at 30 °C to a density of OD_600_ = 1.0 before autophagy was induced by the addition of 0.2 μM rapamycin (LC Laboratories, R-5000). The strains prepared in this study will be distributed upon request.

### Vacuole isolation

Yeast vacuoles were isolated from whole cells as previously described^26,52^ with some modifications. Cells were grown in rich medium or SDCA-uracil to a density of OD_600_ = 1.0 before supplementation of 0.2 μM rapamycin and incubation for further 1, 2, or 3 h. Spheroplasts were prepared by incubation of cells in spheroplast buffer (1.2 M sorbitol, 50 mM Tris-HCl pH 7.5, 50 mM 2-mercaptoethanol, and 5 U/ml Zymolyase 100 T [nacalai tesque, 07665-55]) for 30 min. Spheroplasts were then collected by centrifugation, washed with wash buffer (1.2 M sorbitol and 50 mM Tris-HCl pH 7.5), resuspended in ice-cold buffer A (12% w/v Ficoll 400, 0.1 mM MgCl_2_, 10 mM MES-Tris pH 6.9, and 1 × complete, EDTA-free protease inhibitor cocktail [Roche, 5056489001]), and homogenized with Dounce homogenizer on ice. All the subsequent handling was performed on ice. The lysate was next transferred to an ultracentrifuge tube and buffer B (8% w/v Ficoll 400, 0.1 mM MgCl_2_, and 10 mM MES-Tris pH 6.9) was layered on top, and centrifuged at 72,000 × *g* in a P28S swinging bucket rotor (Hitachi Koki) for 30 min at 4 °C. The top, white layer (crude vacuole) was collected in new ultracentrifuge tube and resuspended in buffer B. The crude vacuole was underlaid with buffer B′ (4% w/v Ficoll 400, 0.1 mM MgCl_2_, and 10 mM MES-Tris pH 6.9) and subjected to a further round of centrifugation at 72,000 × *g* in a P40ST swinging bucket rotor (Hitachi Koki) for 30 min at 4 °C. The top layer was collected (the ‘vacuole fraction’) and RNA or protein were isolated immediately. The vacuole fraction used in analysis in Figs. 2d, 3, 4c, 4d, and Supplementary Fig. 5 was treated with RNase I (Lucigen, N6901K). The RNase I was inactivated by dithiothreitol (DTT) for 20 min at 70 °C before RNA isolation.

### Vacuole recovery estimation

The activity of a vacuolar enzyme, alkaline phosphatase (ALP), was employed as previously described^53^ to biochemically determine the yield of vacuoles recovered from cells by ultracentrifugation. Five microliter of vacuole fraction or whole-cell lysate were suspended in 500 µl of assay buffer (250 mM Tris-HCl pH 9.0, 10 mM MgSO_4_, and 10 µM ZnSO_4_). The reaction was initiated by addition of 50 µl of 55 mM α-naphthyl phosphate. After incubation for 10 min at 30 °C, 500 µl of 2 M glycine-NaOH (pH 11.0) was added to stop the reaction. The fluorescence intensity was measured at 345 nm excitation and 472 nm emission. The vacuole recovery was calculated by the fluorescence intensity of the vacuole fraction normalized to that of whole-cell lysate.

### Isolation, electrophoresis, northern blotting, and qPCR of RNA

RNAs from yeast lysate and isolated vacuoles were extracted by TRIzol reagent (Thermo Fisher Scientific, 15596018) following the manufacturer’s instructions. rRNA subtraction was performed using the Ribominus Transcriptome Isolation Kit (Yeast) (Invitrogen, K155003) according to the manufacturer’s instructions. For electrophoresis, the extracted RNAs were separated on denaturing formaldehyde agarose gel (1% agarose, 1 × MOPS buffer pH 7.0 [20 mM MOPS, 5 mM NaOAc, and 1 mM EDTA], and 2% formaldehyde) and stained with GelRed (1:3300 dilution in water) (Biotium, 41003). For Northern blotting, digoxigenin (DIG) conjugated probes were prepared by PCR DIG Probe Synthesis Kit (Roche, 11636090910) according to the manufacturer’s instructions. The probe was hybridized using PerfectHyb Plus (Sigma–Aldrich, H7033) for 2 h at 68 °C. A DIG-labeled *HOM2* probe was prepared from isolated yeast genomic DNA using 5′-CGTTGGTCAACGTTTCATTCTGTTGTTG-3′ and 5′-AGTGGTCAAAGCATCAATAGGACCG-3′ oligonucleotides. DIG-labeled *ARO2* probe was prepared using 5′-CACCACATATGGTGAATCGCATTGTAAGT-3′ and 5′-GTTCAACAGATGCTGAAATTCAGGATCG-3′ oligonucleotides. Anti-digoxigenin-AP (Roche, 11093274910, 1:10000) and CDP-Star (Roche, 11685627001) were used for signal detection. Chemiluminescence images were acquired using a FUSION-FX7 (Vilber-Lourmat) imaging systems. For qPCR, cDNAs were prepared using the PrimeScript RT reagent kit with gDNA eraser (TAKARA, RR047). A random hexamer was used for cDNA synthesis. Subsequent qPCR was conducted using TB Green Premix Ex Taq II (Tli RNase H Plus) (TAKARA, RR820) with the mRNA-specific primers listed in Supplementary Table 3. Serial dilutions of cDNA were used for qPCR calibrations. Melting-curve analyses confirmed the amplification of a single product for each mRNA. The proportion of each mRNA species in the vacuole (the “proportion in vacuoles”) was calculated by the value of the vacuole fraction normalized to that of the total lysate, considering the vacuole recovery rate as described above.

### Sequencing of RNAs isolated from total cell lysates and vacuolar fractions

Two hundred nanogram of RNA was isolated from total cell lysates or vacuolar fractions using TRIzol reagent (Thermo Fisher Scientific, 15596018) for extraction and and Direct-zol RNA MiniPrep Kit (Zymo Research, R2052) for purification, both according to the manufacturer’s instructions. After rRNA depletion was performed using the Ribo-Zero rRNA Removal Kit (yeast) (Illumina, MRZY1324), libraries were prepared using the TruSeq RNA Library Preparation Kit v2 (Illumina, RS-122-2001), according to the manufacturer’s instructions. The multiplexed libraries were sequencing on a HiSeq 4000 sequencer (Illumina) for single-end 50 bp.

### Ribosome profiling

Thirty OD_600_ units of yeast cells treated with 0.2 μM rapamycin for 1, 2, and 3 h or without rapamycin treatment (0 h) were collected by filtration. Ribosome profiling libraries were prepared by modifying a previously described method^54,55^. The collected cells with drip of ribosome profiling lysis buffer (20 mM Tris-HCl pH 7.5, 150 mM NaCl, 5 mM MgCl_2_, 1 mM DTT, 1% Triton X-100, 100 µg/ml cycloheximide [Sigma–Aldrich, C-7698], and 100 µg/ml chrolamphenicol [Wako, 032-19451]) were ground using Multi-Beads Shocker (Yasui Kikai) with chamber and ball precooled with liquid nitrogen. The cell lysate was centrifuged at 3000 × *g* for 5 min to remove cellular debris. The supernatant was collected to a fresh tube and centrifuged at 20,000 × *g* for 10 min to remove nuclei and other cellular debris. The supernatant containing 20 µg of total RNA was treated with 0.5 U/µg of RNase I (Lucigen, N6901K) at 25 °C for 45 min. A linker oligonucleotide 5′-(Phos)NNNNNIIIIIAGATCGGAAGAGCACACGTCTGAA(ddC)-3′, where (Phos) and (ddC) indicate 5′ phosphorylation and a terminal 2′, 3′-dideoxycytidine, respectively, was used. The Ns and Is indicate random barcode (i.e., unique molecular index, UMI) for eliminating PCR duplication and multiplexing barcode, respectively. The linkers were preadenylated with 5′ DNA Adenylation Kit (NEB, E2610S) and then purified using Oligo Clean & Concentrator (Zymo Research, D4060). The linker ligated footprints were reverse transcribed with a primer 5′-(Phos)NNAGATCGGAAGAGCGTCGTGTAGGGAAAGAG(iSp18)GTGACTGGAGTTCAGACGTGTGCTC-3′, where iSp18 stands for an internal 18-atom hexa-ethyleneglycol spacer, and circularized by CircLigase II (Lucigen, CL9025K). The cDNA was PCR-amplified using primers 5′- AATGATACGGCGACCACCGAGATCTACACTCTTTCCCTACACGACGCTC-3′ and 5′-CAAGCAGAAGACGGCATACGAGATATCACGGTGACTGGAGTTCAGACGTGTG-3′.

For library preparation of RNA-Seq, total RNA was extracted from the same lysate for ribosome profiling using TRIzol LS reagent (Thermo Fisher Scientific, 10296010) and Direct-zol RNA MiniPrep Kit (Zymo Research, R2052). Libraries were prepared using the TruSeq RNA Library Preparation Kit v2 (Illumina, RS-122-2001) as described in the product documentation.

Following multiplexing, ribosome profiling and RNA-Seq libraries were subjected to single-end 50 bp sequencing on a HiSeq 4000 sequencer (Illumina).

### Data analysis

#### RNA-seq

The adapter sequence was trimmed using the FASTX_clipper, a part of the FASTX-Toolkit (https://www.scirp.org/(S(351jmbntvnsjt1aadkposzje))/reference/ReferencesPapers.aspx?ReferenceID=1037549). Bowtie2^56,57^ was used to map the clipped reads to yeast rRNAs and capture unaligned reads. The unaligned reads were mapped to the S288C *S. cerevisiae* reference genome using Tophat^58^ and counted by HTSeq^59^. All genes in Saccharomyces Genome Database (SGD) (https://www.yeastgenome.org)^60^ with reliable sequence annotations and ≥10 read counts were analyzed by DESeq^61^ to calculate vacuolar fraction and whole-cell reads. mRNA enrichment in vacuoles was calculated with DESeq using a generalized linear model to normalize overall mRNA expression change in total lysate after rapamycin treatment. Vacuole-enriched and -depleted mRNAs were defined as transcripts with accumulation in vacuoles at more or less than two-fold vacuolar enrichment, respectively, and with a *q* value below 0.01. Gene ontology analysis was performed using the iPAGE^62^ web interface (https://tavazoielab.c2b2.columbia.edu/iPAGE/).

#### Ribosome profiling

Reads mapped to yeast noncoding RNAs (rRNAs, tRNAs, snRNAs, snoRNAs, and mitochondrial rRNAs) were excluded. The remaining reads were then aligned to the S288C yeast genome with STAR^63^. PCR-duplicated reads with the same UMI in the linker sequence were removed from downstream analysis. Footprints with 26–30 nt were used for analysis. The distance of the A-site from the 5′-end of the reads was estimated for each footprint length as 15 nt for 26–28 nt reads and 16 nt for 29 and 30 nt reads. The change in ribosome association on mRNAs throughout time course of rapamycin treatment was calculated with DESeq using a generalized linear model to normalize overall mRNA expression change after rapamycin treatment. Polarity scores were calculated as a read distribution bias along CDS, as previously described^42^. The reads located at the A-site in first and last 15 nt of CDS were excluded from analysis. Genes with ≥ 64 read counts in CDS were analyzed.

Statistical analyses of all data were performed using R software (https://www.r-project.org/) in the RStudio interface (https://www.rstudio.com/).

### Preparation of in vitro transcribed 4-thioUTP-labeled spike-in RNA

A PCR fragment encoding *Renilla reniformis* luciferase was amplified from the psiCHECK plasmid using 5′-TAATACGACTCACTATAGG-3′ and 5′-CACACAAAAAACCAACACACAG-3′ oligonucleotides (Supplementary Table 3). RNA was transcribed from this fragment using the T7-Scribe Standard RNA IVT Kit (CELLSCRIPT, C-AS2607) and 4-thioUTP. After transcription, a cap structure and poly(A) tail were added using the ScriptCap m^7^G Capping System (CELLSCRIPT, C-SCCE0625) and A-Plus Poly(A) polymerase Tailing Kit (CELLSCRIPT, C-PAP5104H), respectively.

### 4-thiouracil labeling experiment

The 4-thiouracil (4-thioU) metabolic labeling and purification of 4-thioU-labeled RNA was performed by modifying a previously described method^64^. Briefly, cells were grown in synthetic defined medium with low concentration of uracil (SD-low uracil: 0.2% Yeast Synthetic Drop-out Medium Supplements without uracil [Sigma–Aldrich, Y1501], 0.17% Difco yeast nitrogen base without amino acids and ammonium sulfate, 0.0013% uracil, and 2% glucose) overnight (yielding a density of OD_600_ 2–4). Next, cells were diluted into the SD-low uracil medium at OD_600_ 0.1. Upon growth of cells to OD_600_ 0.6, cells were incubated in the presence of 1 mM of 4-thioU (Sigma–Aldrich, 440736) for 2 h before washing out into fresh synthetic defined medium (SD: 0.2% Yeast Synthetic Drop-out Medium Supplements without uracil [Sigma–Aldrich, Y1501], 0.17% Difco yeast nitrogen base without amino acids and ammonium sulfate, 0.002% uracil, and 2% glucose) without 4-thioU. Cells were then treated with rapamycin for 1 h and collected immediately.

RNAs were extracted by a hot phenol method, as described previously^13^. Frozen cells were resuspended in 400 µl of AE buffer (50 mM sodium acetate and 10 mM EDTA pH 5.0) with 1% SDS, and placed at 65˚C. Immediately, cells were resuspended in 500 µl of AE buffer-saturated hot phenol and homogenized using 0.5 mm zirconia beads (Yasui Kikai, YZB05) and FastPrep-24 (MP Biomedicals, 6004-500) with a setting of 30 s at 5.5 m/s for four times. The samples were centrifuged at 15,000 × *g* for 10 min and the RNA were extracted with ANE buffer (10 mM sodium acetate, 100 mM NaCl, and 2 mM EDTA)-saturated phenol:chloroform and then chloroform:isoamyl alcohol. Subsequently the RNA was precipitated with ethanol. Ten microgram of total RNA was mixed with 5 ng of in vitro transcribed 4-thioU-labeled *Renilla reniformis* luciferase mRNA, biotinylated using 0.2 mg/ml of EZ-Link HPDP-Biotin (Thermo Fisher Scientific, 21341) in 10 mM Tris-HCl pH 7.5 and 1 mM EDTA for 2 h at 23 °C in the dark, and then precipitated with isopropanol. The biotinylated RNAs were incubated with Dynabeads MyOne Streptavidin C1 (Thermo Fisher Scientific, DB6500) for 15 min. The beads were washed with buffer 1 (100 mM Tris-HCl pH 7.4, 0.5 M EDTA, and 5 M NaCl) prewarmed to 65 °C once, buffer 2 (100 mM Tris-HCl pH 7.4, 0.5 M EDTA, and 10% SDS) once, and then 10% buffer 1 twice. The RNAs were eluted from the streptavidin beads with 5% β-mercaptoethanol for 5 min at room temperature and then for 10 min at 65 °C, and subsequently precipitated with isopropanol.

4-thioU incorporation into RNA was evaluated by a dot-blot assay, as previously described^65^ with some modifications. Biotinylated RNA was spotted onto the positively charged nylon membrane and then crosslinked to the membrane by exposure to UV light. The membrane was incubated with 10 × blocking solution (125 mM NaCl, 9 mM Na_2_HPO_4_, 7 mM NaH_2_PO_4_, and 10% SDS) for 20 min. Anti-streptavidin-HRP (Abcam, ab7403, 1:5000) was added to the solution and incubated for 10 min. The membrane was washed twice by 1 × blocking solution for 10 min and twice by wash solution (10 mM Tris-Base, 10 mM NaCl, and 1.05 mM MgCl_2_, pH 9.5) for 5 min. Chemiluminescence images detected with Femtoglow HRP Substrate (Michigan Diagnostics, 21008) were acquired by a FUSION-FX7 (Vilber-Lourmat) imaging system.

### Western blotting

Western blotting was performed with 0.2 OD_600_ units of cells harvested for sample preparation. Frozen cells were treated with 10% trichloroacetic acid (TCA) for 5 min on ice. After TCA was discarded by centrifugation, the pellet was washed with ice-cold acetone and resuspended in 1 × sample buffer (75 mM Tris-HCl pH 6.5, 10% glycerol, 25 mM DTT, and 0.6% SDS). The samples were homogenized using 0.5 mm zirconia beads (Yasui Kikai, YZB05) and FastPrep-24 (MP Biomedicals, 6004-500) with a setting of 60 s at 6.0 m/s, and then incubated at 65˚C for 10 min. Samples were separated by SDS-PAGE followed by Western blotting. Anti-FLAG (Sigma–Aldrich, F3165, 1:1000), anti-Pho8 (Abcam, ab113688, 1:1000), anti-Ape1 (1:5000)^66^, anti-β-actin (Wako, 010-27841, 1:500), anti-Dpm1 (Invitrogen, A6429, 1:1000), anti-Gsp1 (ImmuQuest, IQ241, 1:10000), anti-Van1 (a gift from Koji Yoda, 1:3000), and anti-GFP (Roche, 11814460001, 1:1000) were used as primary antibodies. Chemiluminescence was raised by Femtoglow HRP Substrate (Michigan Diagnostics, 21008) and blots were visualized using LAS-4000 (GE Healthcare) or FUSION-FX7 (Vilber-Lourmat) imaging systems.

### Polysome analysis

Two hundred OD_600_ units of yeast cells grown in rich medium with or without rapamycin were treated with 0.1 mg/ml of cycloheximide (Sigma–Aldrich, C-7698) for 5 min on ice and collected by centrifugation. The cell pellet was flash-frozen in liquid nitrogen, ground with a mortar and pestle precooled on liquid nitrogen, and then resuspended in lysis buffer (20 mM HEPES-KOH pH 7.4, 100 mM potassium acetate, and 2 mM magnesium acetate). The cell lysate was centrifuged at 1100 × *g* for 10 min to remove cellular debris. The supernatant was collected to a fresh tube and centrifuged at 9100 × *g* for 10 min to remove nuclei and other cellular debris. Supernatants containing 100 µg of total cellular RNA were layered on top of sucrose gradients (10–50% sucrose in 10 mM Tris-acetate pH 7.4, 70 mM ammonium acetate, and 4 mM magnesium acetate) prepared in ultracentrifuge tubes (Hitachi Koki) using a Gradient Master (BIOCOMP). Samples were then centrifuged at 150,000 × *g* in a P40ST rotor (Hitachi Koki) for 2.5 h at 4 °C. Gradients were fractionated using Piston Gradient Fractionator (BIOCOMP). Continuous absorbance was measured at 254 nm using a single path UV-1 optical unit (Biomini UV-monitor, ATTO).

### Plasmid construction

Plasmids used in this study are listed in Supplementary Table 2 and will be distributed upon request.

#### *pRS416*-CYC1t URA3 CEN (*pSM21*)

A DNA fragment containing the CYC terminator was amplified by PCR from pRS416*-GPDp-CYC1t URA3 CEN*^67^ and inserting this fragment into pRS416-*URA3 CEN* using XhoI and KpnI sites.

#### *pRS416*-HOM2-FLAG-CYC1t (*pSM37*) and *pRS416*-ARO2-FLAG-CYC1t (*pSM75*)

DNA fragments containing the *HOM2* or *ARO2* genes spanning a region from 500 nt upstream of the initiation codon (containing promoter and 5′ UTR sequences) to the 3′ UTR were amplified by PCR from genomic DNA. These DNA fragments were inserted to pRS416*-CYC1t URA3 CEN* using SacI and XhoI sites. A FLAG-tag encoding sequences was then inserted into the region upstream of stop codon by inverse PCR.

#### *pRS416*-stem-loop-HOM2-FLAG-CYC1t (*pSM38*)

A DNA fragment containing BamHI and SalI sites were inserted into 10 nucleotides upstream of the start codon of *HOM2* in pRS416*-HOM2-FLAG-CYC1t* by inverse PCR. Two DNA oligonucleotides (5′-GATCCCCCGGAGATCCCGCGGTTCGCCGCGGGCGTACG-3′ and 5′-TCGACGTACGCCCGCGGCGAACCGCGGGATCTCCGGGG-3′) were annealed and then inserted into the pRS416*-HOM2-FLAG-CYC1t* using BamHI and SalI sites. See also Supplementary Table 3 for the list of primers used for this construction.

#### *pRS416*-stem-loop-ARO2-FLAG-CYC1t (*pSM76*)

A DNA fragment containing the ORF and 3′ UTR of *ARO2* was amplified by PCR from genomic DNA and then inserted into pRS416*-stem-loop-HOM2-FLAG-CYC1t* using SalI and XhoI sites. A PCR-amplified DNA fragment containing the 500 nucleotides upstream of the *ARO2* initiation codon was also prepared from genomic DNA and inserted into the vector using the SacI and BamHI sites.

#### *pRS416*-HOM2 TTG-FLAG-CYC1t *and pRS416*-HOM2 TAC-FLAG-CYC1t (*pSM60 and 61*)

The start codon of *HOM2* encoded on pRS416*-HOM2-FLAG-CYC1t* was mutated to TTG or TAC by inverse PCR.

#### *pRS416*-HOM2 5′ UTR-GFP-HOM2 3′ UTR-CYC1t *(pSM27)*

A DNA fragment containing *GFP* was inserted into pRS416*-stem-loop-HOM2-FLAG-CYC1t* using BamHI and XhoI sites (pRS416*-HOM2 5*′ *UTR-GFP-CYC1t*). Then, a DNA fragment containing the *HOM2* 3′ UTR was amplified by PCR from genomic DNA and inserted into the vector at XhoI site.

#### *pRS416*-HOM2 5′ UTR-HOM2 ORF-PIG2 3′ UTR-CYC1t *(pSM19)*

A DNA fragment spanning the promoter and the ORF region of *HOM2* was amplified by PCR from genomic DNA as a template and inserting this fragment into pRS416*-CYC1t URA3 CEN* using SacI and XhoI sites. A genome-derived DNA fragment containing the *PIG2* 3′ UTR was then amplified by PCR and inserted into the vector using the XhoI site.

#### *pRS416*-APL5 5′ UTR-HOM2 ORF-FLAG-HOM2 3′ UTR-CYC1t *and pRS416*-ASG1 5′ UTR-HOM2-FLAG-HOM2 3′ UTR-CYC1t *(pSM65 and 66)*

These plasmids were prepared by amplifying a DNA fragment from genomic DNA encoding the region 500 nucleotides upstream of the initiation codon for each gene and inserting each into pRS416*- stem-loop-HOM2-FLAG-CYC1t* using SacI and SalI sites.

### Reporting summary

Further information on research design is available in the Nature Research Reporting Summary linked to this article.

## Supplementary information

Supplementary information

Reporting Summary

## Data Availability

RNA-Seq and ribosome profiling data are deposited in NCBI as GEO: GSE149016. Custom codes used for this study will be provided upon request. Source data are provided with this paper.

## Source data

Source Data
