# Supplementary material for: Selectivity of mRNA degradation by autophagy in yeast
Source: Nat Commun. 2021 Apr 19;12:2316. doi: 10.1038/s41467-021-22574-6 (PMC8055698; doi:10.1038/s41467-021-22574-6)
Supplement: Supplementary file 1 — Supplementary information [file 41467_2021_22574_MOESM1_ESM.pdf]

Supplementary Information for:

Shiho Makino, Tomoko Kawamata, Shintaro Iwasaki, and Yoshinori Ohsumi

Selectivity of mRNA degradation by autophagy in yeast

Supplementary Fig. 1 - 9

Supplementary Table 1: *Saccharomyces cerevisiae* strains used in this study

Supplementary Table 2: Plasmids used in this study

Supplementary Table 3: Oligonucleotides used in this study

Supplementary Fig. 1

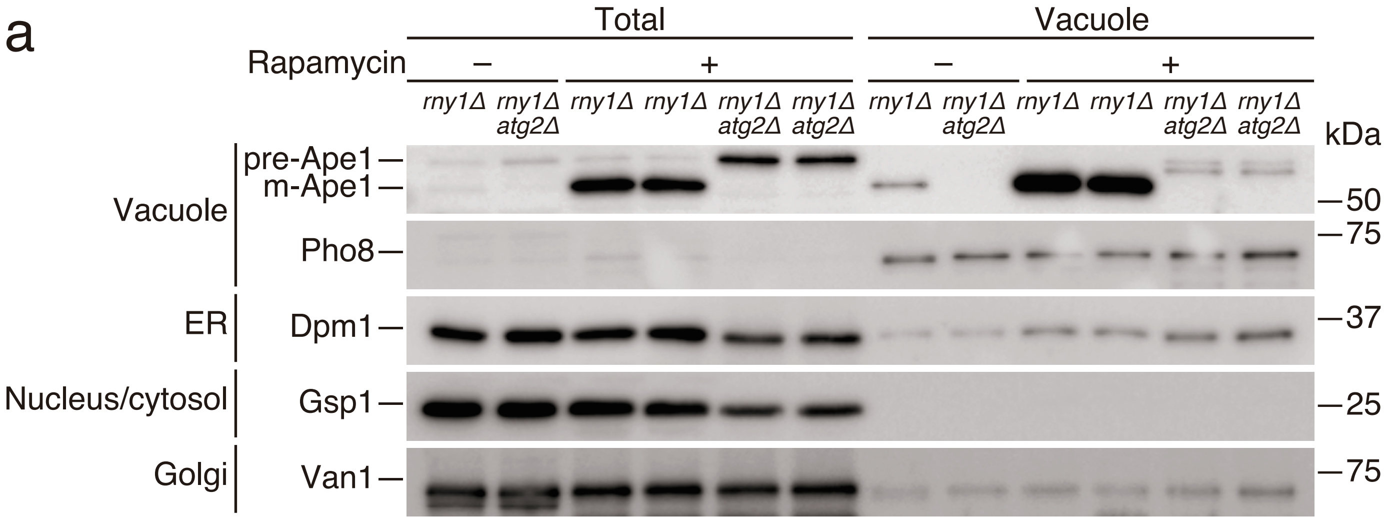

Supplementary Fig. 1. Confirmation of isolated vacuole fraction purity

(a) Western blotting of whole cell lysate and vacuolar fraction samples for RNA-seq using antibodies for vacuole, ER, nucleus/cytosol, and Golgi marker proteins (– Rapamycin samples: $n = 1$; + Rapamycin samples: $n = 2$ independent experiments).

Supplementary Fig. 2

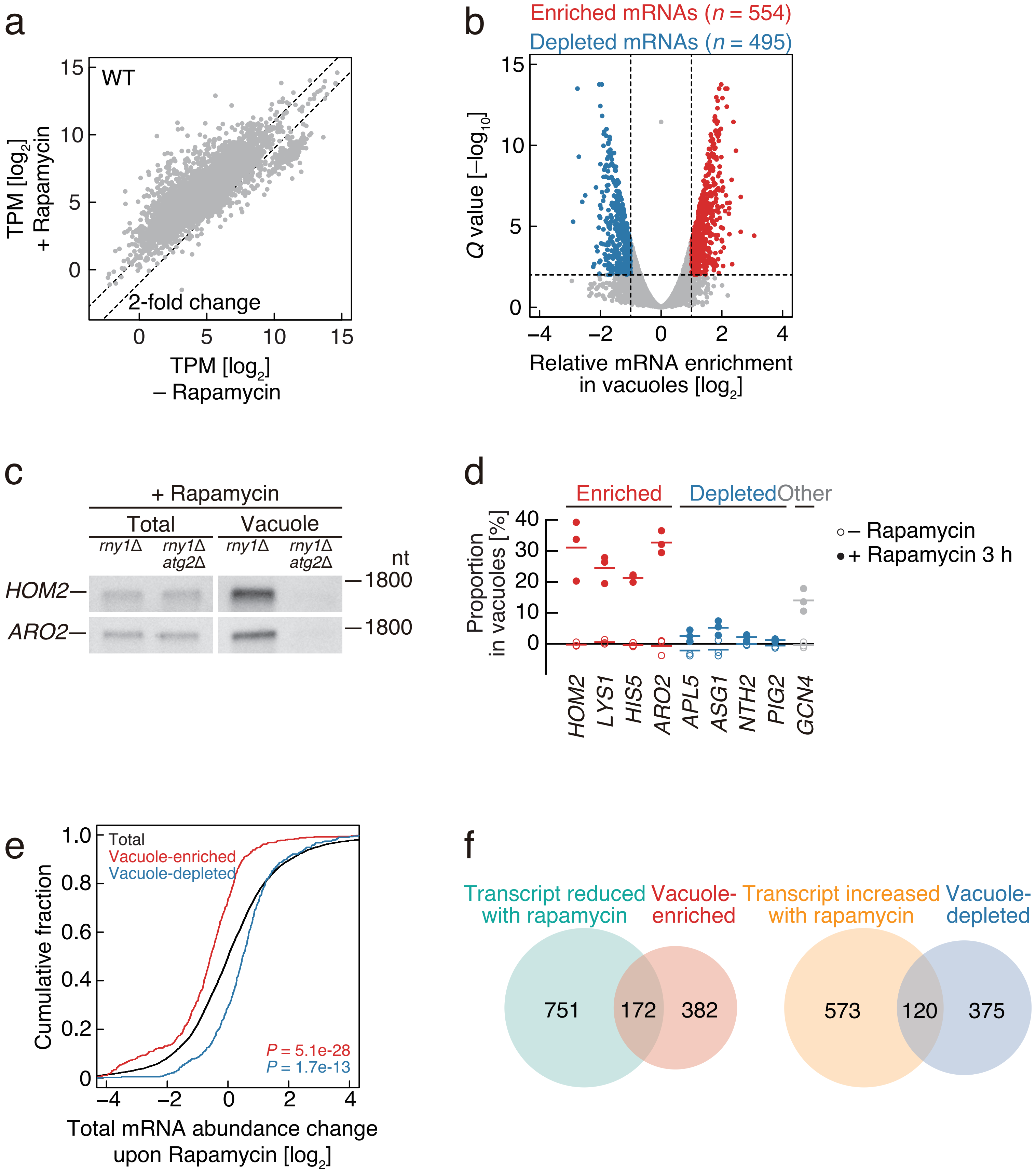

Supplementary Fig. 2. Quantification of mRNA in vacuoles

(a) Scatter plot for mRNA abundance (transcript per million, TPM) in wild-type (WT) cells with or without rapamycin treatment. The dashed lines represent 2-fold change in TPM.

(b) Volcano plot for rapamycin-induced mRNA delivery into vacuoles. Vacuole-enriched (log_2-fold change ≥ 1 and q value < 0.01) and depleted (log_2-fold change ≤ -1 and q value < 0.01) mRNAs were highlighted in red and blue.

(c) Northern blotting for *HOM2* and *ARO2* mRNA in whole cell lysate and vacuolar fraction samples. Representative results from two independent experiments are shown.

(d) The fraction of mRNAs, which are representatively analyzed in this study (shown in Fig. 2c), recovered from the vacuolar fraction in comparison to whole cell lysates in *rny1*Δ cells (subtracting those of *rny1*Δ*atg2*Δ cells) following 3 h rapamycin treatment. Data present mean (line) and individual results (points, without Rapamycin (open circles); with Rapamycin treatment (filled circles)) of three independent experiments.

(e) Cumulative distribution of vacuole-enriched (red) and -depleted (blue) mRNAs (defined in Fig. 2a) in WT cells of total mRNA abundance following 3 h rapamycin treatment. Significance was calculated by unpaired two-sided Mann-Whitney *U*-test.

(f) Venn diagrams showing the overlap between vacuole-enriched mRNAs and transcripts reduced (left) or vacuole-depleted mRNAs and transcripts increased (right). mRNAs significantly changed in WT cells following 3 h rapamycin treatment were classified as “increased” (log_2-fold change ≥ 1 and q value < 0.01) or “reduced” (log_2-fold change ≤ -1 and q value < 0.01).

Supplementary Fig. 3

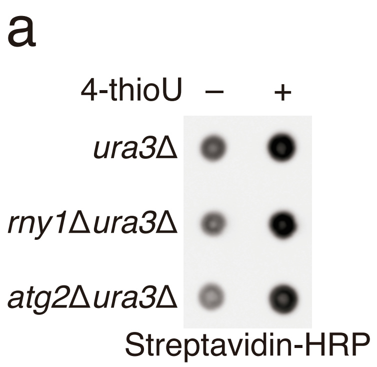

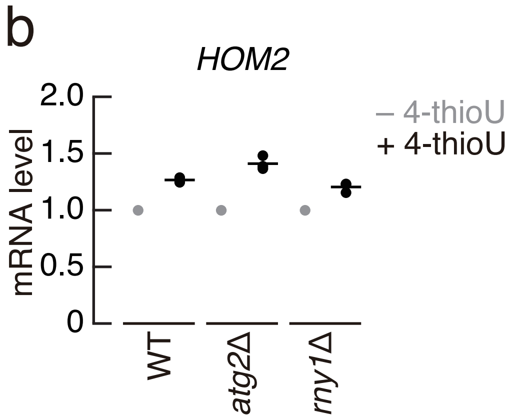

Supplementary Fig. 3. RNA labeling with 4-thiouracil (4-thioU)

(a) Dot blot analysis of 4-thioU labeled RNA sampled immediately following the addition of rapamycin. Blots were probed using streptavidin conjugated to HRP. Representative results from three independent experiments are shown.

(b) *HOM2* mRNA abundance following a 4-thioU labeling (+ 4-thioU) or no 4-thioU labeling (– 4-thioU) immediately after the addition of rapamycin. Data were normalized by setting mRNA abundance in – 4-thioU samples to 1 in each strain. Data present mean (line) and individual results (points) of three independent experiments.

Supplementary Fig. 4

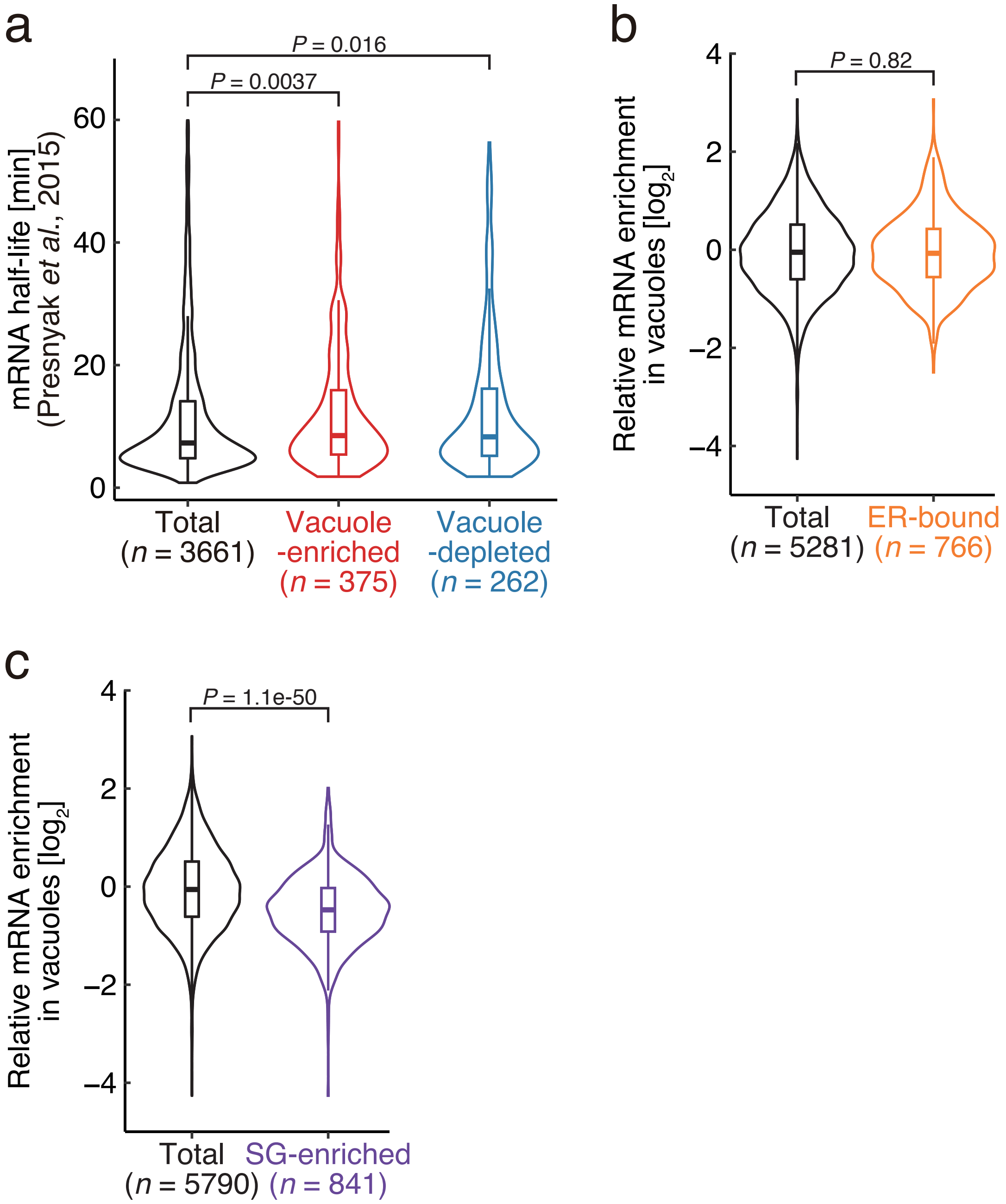

Supplementary Fig. 4. Steady-state half-life, ER association, and stress granular localization do not correlate with vacuolar delivery of mRNAs

(a) Violin plots of vacuole-enriched (red) and depleted (blue) mRNAs (defined in Fig. 2a) in comparison to steady-state mRNA half-life [19].

(b and c) Violin plots of ER-bound (b, orange) and stress granule (SG)-enriched mRNAs (c, purple) in comparison to relative mRNA enrichment in vacuoles by rapamycin treatment.

The median, IQR, and 1.5 IQR are represented by solid line, box, and whiskers, respectively. Significance was determined using unpaired two-sided Mann-Whitney *U*-test.

Supplementary Fig. 5

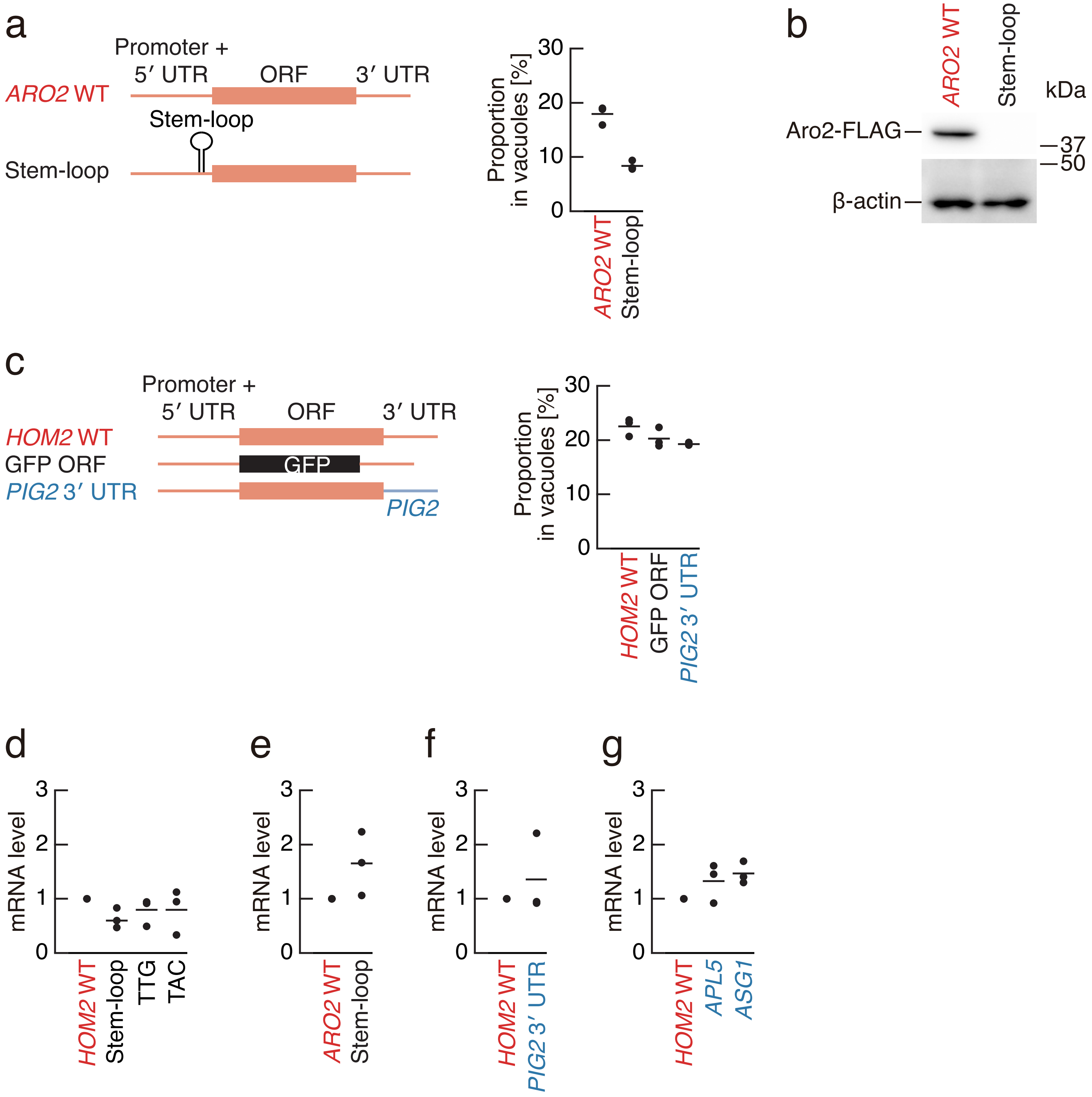

Supplementary Fig. 5. Translation of reporter mRNAs is important for their vacuolar delivery

(a) The fraction of an alternative vacuole-enriched mRNA, *ARO2*, recovered from vacuole fraction following 3 h rapamycin treatment. Data was analyzed as described in Fig. 2d. To block translation, an inhibitory stem-loop was inserted immediately before the *ARO2* initiation codon.

(b) Protein levels of *ARO2* mRNAs (shown in a) were analyzed by Western blotting. Representative results from three independent experiments are shown.

(c) The GFP ORF and *PIG2* 3′ UTR was switched with the *HOM2* ORF and 3′ UTR, respectively, and analyzed as described in Fig. 2d.

(d-g) mRNA abundance of the cell lysates used in Fig. 3b (d), Supplementary Fig. 5a (e), Supplementary Fig. 5c (f), and Fig. 3d (g) after 3 h rapamycin treatment. mRNA level was quantified by qPCR. In f, data for GFP ORF reporter was not shown as qPCR primers hybridizing to *HOM2* ORF could not applied. Data were normalized to mRNA abundance of *HOM2* WT mRNA.

Data in a and c-g present mean (line) and individual results (points) of three independent experiments.

Supplementary Fig. 6

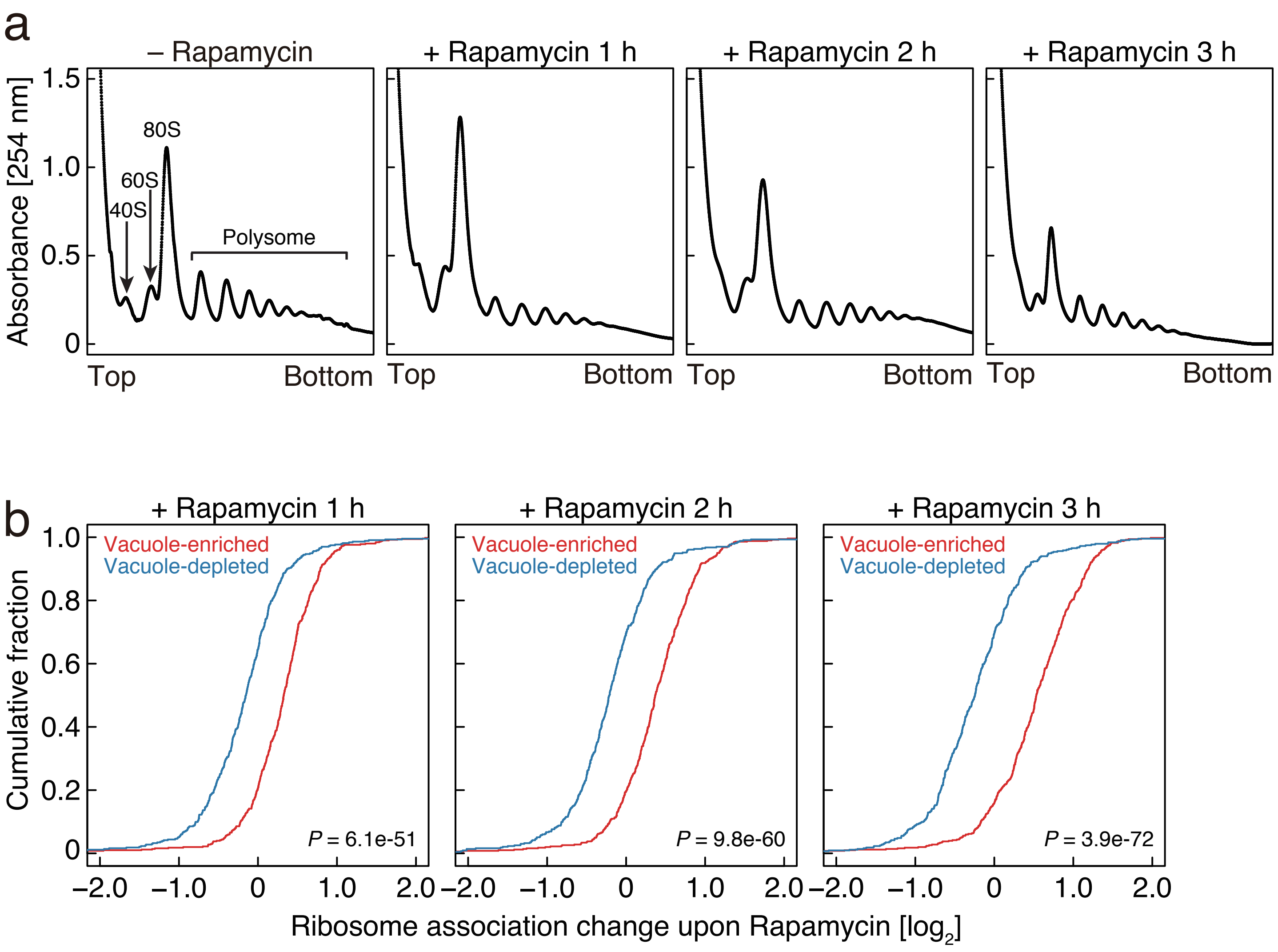

Supplementary Fig. 6. Ribosome profiling reveals the whole cell translatome during rapamycin treatment

(a) Polysome profiles of wild-type cells at 0, 1, 2, and 3 h rapamycin treatment.

(b) Cumulative distribution of vacuole-enriched (red) and -depleted (blue) mRNAs (defined in Fig. 2a) in comparison to change in ribosome association following 1, 2, or 3 h rapamycin treatment. Significance was calculated using unpaired two-sided Mann-Whitney *U*-test.

Supplementary Fig. 7

a

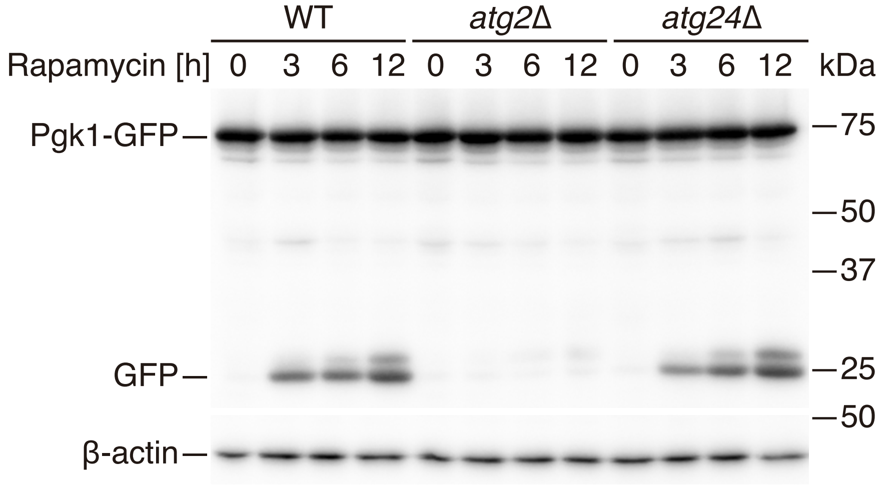

b

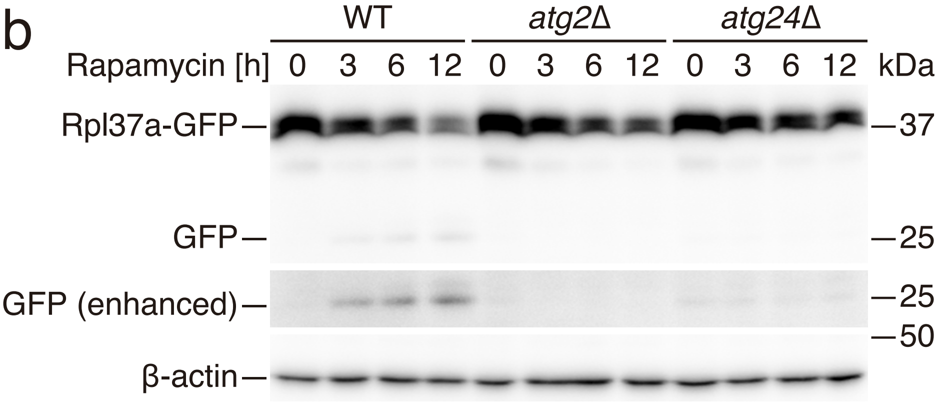

Supplementary Fig. 7. Atg24 is required for autophagic degradation of ribosomes

(a, b) Pgk1-GFP and Rpl37a-GFP degradation were analyzed by Western blotting to determine the vacuole-dependent cleavage of GFP from each chimeric protein. Representative results from two independent experiments are shown.

Supplementary Fig. 8

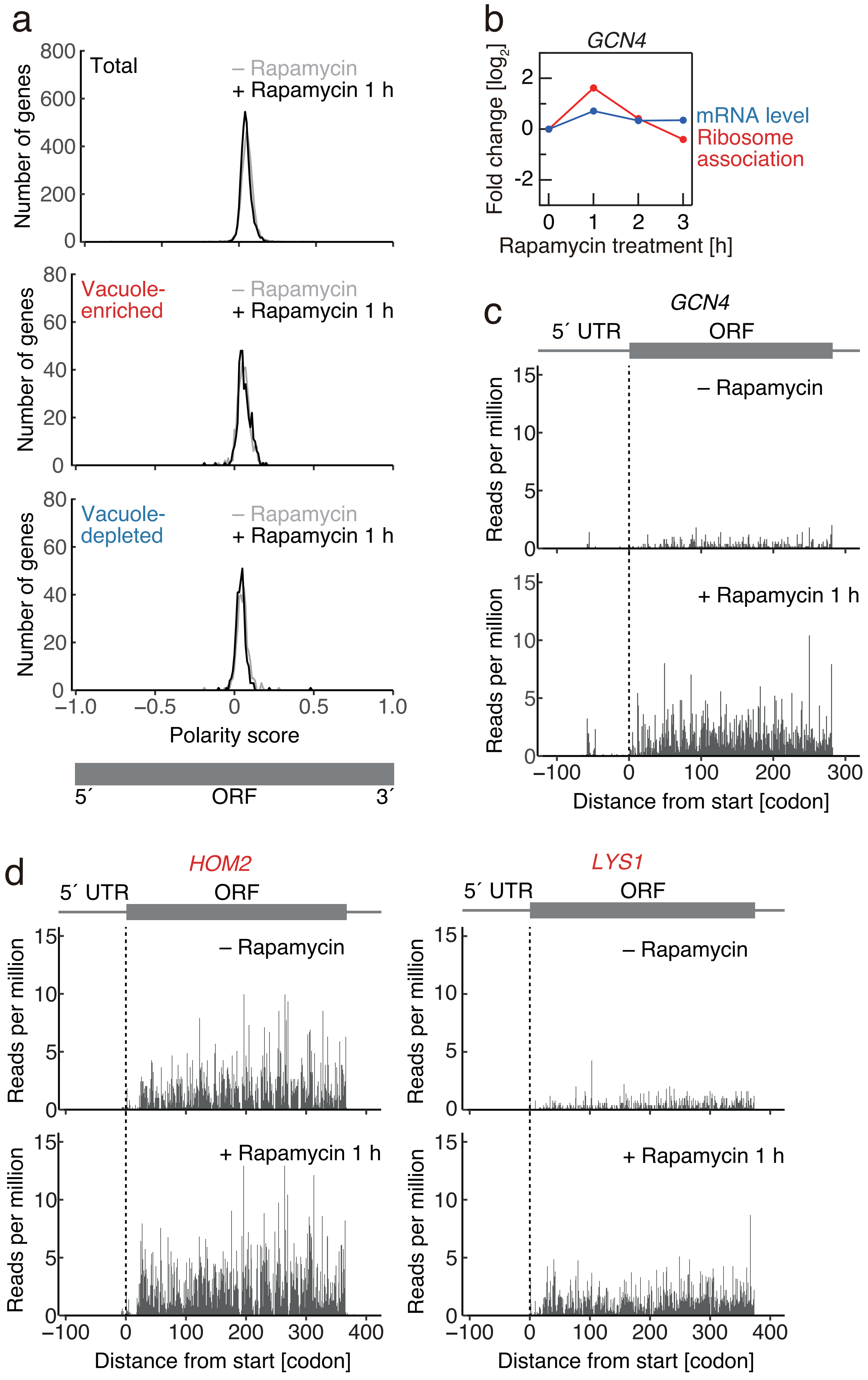

Supplementary Fig. 8. uORF-independent translation regulation of vacuole-enriched mRNAs during TORC1 inhibition

(a) Distribution of polarity scores for 3718 mRNAs from total mRNAs, 375 mRNAs from vacuole-enriched mRNAs, and 329 mRNAs from vacuole-depleted mRNAs with or without rapamycin treatment. A negative score indicates 5′ polarity shift, whereas a positive score indicates 3′ polarity shift from the center of ORF, respectively.

(b) mRNA abundance and ribosome association of *GCN4* mRNA during rapamycin treatment, as determined by ribosome profiling and RNA-Seq.

(c and d) Ribosome footprint distributions for *GCN4* (c) and *HOM2* and *LYS1* (d) mRNAs with or without rapamycin treatment.

Supplementary Fig. 9

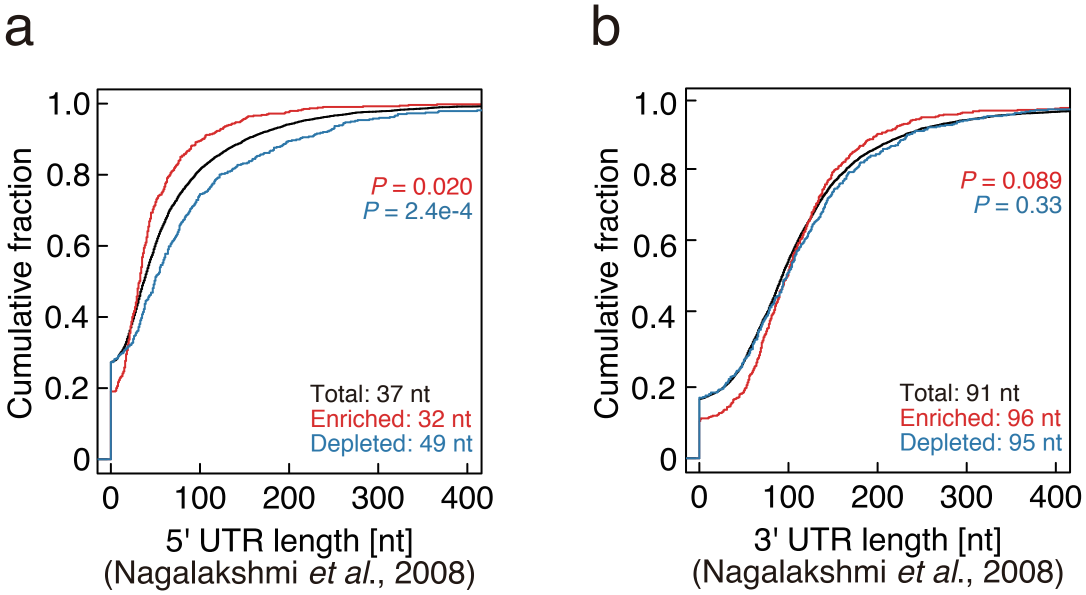

Supplementary Fig. 9. Properties of untranslated region length in vacuole-enriched or -depleted mRNAs

(a, b) Cumulative distribution of vacuole-enriched (red) and -depleted (blue) mRNAs (defined in Fig. 2a) in comparison to 5′ UTR (a) or 3′ UTR length (b). The median length of each mRNAs is shown. Significance was calculated using unpaired two-sided Mann-Whitney *U*-test.

Supplementary Table 1. ***Saccharomyces cerevisiae*** **strains used in this study**

Strain	Genotype	Source
X2180-1a	*MAT*a *SUC2 mal mel gal2 CUP1*	Yeast Genetic Stock Center
MMY3	X2180-1B; *atg2*Δ::*kanMX6*	Huang *et al*., 2015
MMY204	X2180-1B; *rny1*Δ::*natNT2*	Huang *et al*., 2015
MMY375	X2180-1B; *rny1*Δ::*natNT2, atg2*Δ::*kanMX6*	Huang *et al*., 2015
MMY274	X2180-1B; *ura3*Δ::*natNT2*	This study
SMY57	X2180-1B; *ura3*Δ::*kanMX6, rny1*Δ::*natNT2*	This study
YSM122	X2180-1B; *ura3*Δ::*natNT2, atg2*Δ::*hphNT1*	This study
YSM132	X2180-1B; *rny1*Δ::*natNT2*, *ura3*Δ::*kanMX6*, *hom2*Δ::*hphNT1*	This study
YSM164	X2180-1B; *rny1*Δ::*natNT2*, *ura3*Δ::*kanMX6*, *aro2*Δ::*hphNT1*	This study
YSM166	X2180-1B; *rny1*Δ::*natNT2*, *atg24*Δ::*hphNT1*	This study
HSY199	X2180-1B; *PGK1-GFP*:: *natNT2*	This study
HSY200	X2180-1B; *PGK1-GFP*:: *natNT2*, *atg2*Δ:: *kanMX6*	This study
HSY201	X2180-1B; *PGK1-GFP*:: *natNT2*, *atg24*Δ::*hphNT1*	This study
SMY36	X2180-1B; *RPL37A-GFP*:: *kanMX6*	Kawamata *et al*., 2017
SMY41	X2180-1B; *RPL37A-GFP*:: *kanMX6*, *atg2*Δ:: *natNT2*	Kawamata *et al*., 2017
YSM215	X2180-1B; *RPL37A-GFP*:: *kanMX6*, *atg24*Δ:: *hphNT1*	This study
YSM182	X2180-1B; *rny1*Δ::*natNT2*, *ura3*Δ::*kanMX6*, *hom2*Δ::*hphNT1* pRS416-*HOM2-FLAG-CYC1t*	This study
YSM181	X2180-1B; *rny1*Δ::*natNT2*, *ura3*Δ::*kanMX6*, *hom2*Δ::*hphNT1*<br>pRS416-*HOM2 5' UTR-GFP-HOM2 3' UTR-CYC1t*	This study
YSM172	X2180-1B; *rny1*Δ::*natNT2*, *ura3*Δ::*kanMX6*, *hom2*Δ::*hphNT1*<br>pRS416- *HOM2 5' UTR-HOM2 ORF-PIG2 3' UTR-CYC1t*	This study
YSM208	X2180-1B; *rny1*Δ::*natNT2*, *ura3*Δ::*kanMX6*, *hom2*Δ::*hphNT1*	This study

	pRS416- *APL5 5′ UTR-HOM2 ORF-FLAG-HOM2 3′ UTR-CYC1t*	
YSM209	X2180-1B; *rny1*Δ::*natNT2*, *ura3*Δ::*kanMX6*, *hom2*Δ::*hphNT1*<br>pRS416- *ASG1 5′ UTR-HOM2-FLAG-HOM2 3′ UTR-CYC1t*	This study
YSM183	X2180-1B; *rny1*Δ::*natNT2*, *ura3*Δ::*kanMX6*, *hom2*Δ::*hphNT1*<br>pRS416-*stem-loop-HOM2-FLAG-CYC1t*	This study
YSM203	X2180-1B; *rny1*Δ::*natNT2*, *ura3*Δ::*kanMX6*, *hom2*Δ::*hphNT1*<br>pRS416-*HOM2 TTG-FLAG-CYC1t*	This study
YSM204	X2180-1B; *rny1*Δ::*natNT2*, *ura3*Δ::*kanMX6*, *hom2*Δ::*hphNT1*<br>pRS416-*HOM2 TAC-FLAG-CYC1t*	This study
YSM223	X2180-1B; *rny1*Δ::*natNT2*, *ura3*Δ::*kanMX6*, *aro2*Δ::*hphNT1*<br>pRS416-*ARO2-FLAG-CYC1t*	This study
YSM224	X2180-1B; *rny1*Δ::*natNT2*, *ura3*Δ::*kanMX6*, *aro2*Δ::*hphNT1*<br>pRS416-*stem-loop-ARO2-FLAG -CYC1t*	This study
HSY430	X2180-1B; *rny1*Δ::*natNT2*, *atg20*Δ::*hphNT1*	This study
HSY432	X2180-1B; *rny1*Δ::*natNT2*, *snx41*Δ::*hphNT1*	This study

Supplementary Table 2. Plasmids used in this study

Plasmid name	Feature	Source
pRS416	*CEN6, URA3*	Mumberg et al., 1995
pRS416*GPDp-CYC1t*	*CEN6, URA3, GPD promoter, CYC1 terminator*	Mumberg et al., 1995
pSM21	pRS416-*CYC1t*	This study
pSM37	pRS416-*HOM2-FLAG-CYC1t*	This study
pSM27	pRS416-*HOM2 5′ UTR-GFP-HOM2 3′ UTR-CYC1t*	This study
pSM19	pRS416-*HOM2 5′ UTR-HOM2 ORF-PIG2 3′ UTR-CYC1t*	This study
pSM65	pRS416-*APL5 5′ UTR-HOM2 ORF-FLAG-HOM2 3′ UTR-CYC1t*	This study
pSM66	pRS416-*ASG1 5′ UTR-HOM2-FLAG-HOM2 3′ UTR-CYC1t*	This study
pSM38	pRS416-*stem-loop-HOM2-FLAG-CYC1t*	This study
pSM60	pRS416-*HOM2 TTG-FLAG-CYC1t*	This study
pSM61	pRS416-*HOM2 TAC-FLAG-CYC1t*	This study
pSM75	pRS416-*ARO2-FLAG-CYC1t*	This study
pSM76	pRS416-*stem-loop-ARO2-FLAG-CYC1t*	This study

Supplementary Table 3. The oligonucleotides used in this study

Name description	Sequence
For qPCR	
LYS1 Fw	5′-AAAACTGCTCCTGTCTGGGTTA-3′
LYS1 Rv	5′-TTTAACACGAGCGCAATGTC-3′
ASG1 Fw	5′-AAACTATCCAGCTTACTGCGTTACA-3′
ASG1 Rv	5′-TCACTAAAAAGGCACTTTCGTTG-3′
HOM2 Fw	5′-CTGGTATCGCCATTGTTTCC-3′
HOM2 Rv	5′-TCAATGGCACATCTTGTTCTCT-3′
APL5 Fw	5′-TCAACTGTATTGTCAAGGGGAAT-3′
APL5 Rv	5′-ATGCCATTGCTGTTTCAAAAT-3′
PIG2 Fw	5′-TTAGGCGAGGAGGAAGACG-3′
PIG2 Rv	5′-TCCTCGGATTCATACTGGAAA-3′
GFP Fw	5′-ATGGTGATGTTAATGGTCACAAA-3′
GFP Rv	5′-AAGTAGCATCACCTTCACCTTCA-3′
ARO2 Fw	5′-AGTACGGTATCAAGGCCTCCT-3′
ARO2 Rv	5′-GGCCAATCGTTTCTCTAGCA-3′
HIS5 Fw	5′-AGGAACAAAACAAGCAGTTATGC-3′
HIS5 Rv	5′-AGGCACAGATTGTCAGCAGTT-3′
NTH2 Fw	5′-GATGAGAATGATGGAACGGTAAC-3′
NTH2 Rv	5′-CCTTGTTTTCCTCAGTTCAGCTA-3′
GCN4 Fw	5′-TCTGCTTCCACTTCTACTGCCAAAC-3′
GCN4 Rv	5′-CAGTTGCCGTTTGTGGAAGAGC-3′
For DIG-labeled probe	
HOM2 Fw	5′-CGTTGGTCAACGTTTCATTCTGTTGTTG-3′
HOM2 Rv	5′-AGTGGTCAAAGCATCAATAGGACCG-3′
ARO2 Fw	5′-CACCACATATGGTGAATCGCATTGTAAGT-3′
ARO2 Rv	5′-GTTCAACAGATGCTGAAATTCAGGATCG-3′
For PCR amplification of Rluc	
Rluc Fw	5′-TAATACGACTCACTATAGG-3′

Rluc Rv	5′-CACACAAAAAACCAACACACAG-3′
For insertion of stem-loop	
Stem-loop 5′	5′-GATCCCCCGGAGATCCCGCGGTTCGCCGCGGGCGTACG-3′
Stem-loop 3′	5′-TCGACGTACGCCCGCGGCGAACCGCGGGATCTCCGGGG-3′
