# Supplementary material for: Selectivity of mRNA degradation by autophagy in yeast
Source: Nat Commun. 2021 Apr 19;12:2316. doi: 10.1038/s41467-021-22574-6 (PMC8055698; doi:10.1038/s41467-021-22574-6)
Supplement: Supplementary file 2 — Reporting Summary [file 41467_2021_22574_MOESM2_ESM.pdf]

nature research

Corresponding author(s): Yoshinori Ohsumi, Shintaro Iwasaki

Last updated by author(s): Mar 8, 2021

Reporting Summary

Nature Research wishes to improve the reproducibility of the work that we publish. This form provides structure for consistency and transparency in reporting. For further information on Nature Research policies, see our Editorial Policies and the Editorial Policy Checklist.

Statistics

For all statistical analyses, confirm that the following items are present in the figure legend, table legend, main text, or Methods section.

n/a	Confirmed	
☐	☒	The exact sample size (*n*) for each experimental group/condition, given as a discrete number and unit of measurement
☐	☒	A statement on whether measurements were taken from distinct samples or whether the same sample was measured repeatedly
☐	☒	The statistical test(s) used AND whether they are one- or two-sided<br>*Only common tests should be described solely by name; describe more complex techniques in the Methods section.*
☒	☐	A description of all covariates tested
☐	☒	A description of any assumptions or corrections, such as tests of normality and adjustment for multiple comparisons
☐	☒	A full description of the statistical parameters including central tendency (e.g. means) or other basic estimates (e.g. regression coefficient) AND variation (e.g. standard deviation) or associated estimates of uncertainty (e.g. confidence intervals)
☐	☒	For null hypothesis testing, the test statistic (e.g. *F*, *t*, *r*) with confidence intervals, effect sizes, degrees of freedom and *P* value noted<br>*Give P values as exact values whenever suitable.*
☒	☐	For Bayesian analysis, information on the choice of priors and Markov chain Monte Carlo settings
☒	☐	For hierarchical and complex designs, identification of the appropriate level for tests and full reporting of outcomes
☒	☐	Estimates of effect sizes (e.g. Cohen's *d*, Pearson's *r*), indicating how they were calculated

Our web collection on statistics for biologists contains articles on many of the points above.

Software and code

Policy information about availability of computer code

Data collection	Ribosome profiling and RNAseq data were collected using Illumina Casava 1.8 software, 3' adapter trimming with FastX-toolkit v0.0.13, library sorting based on sample barcode sequence, and random barcode triming with custom script.<br>rRNA alignment was performed with bowtie2 v2.1.0, and rRNA and other non-coding RNA alignment were performed with STAR v2.7.0a.<br>Reads were aligned with TopHat v2.0.9 or STAR v2.7.0a and quantitated using HTSeq v0.6.1 or custom scripts.
Data analysis	All data were analyzed by DESeq (v1.36.0) with custom scripts using R software (v3.6.0) in the RStudio interface (v1.2.1335).<br>Source codes for ribosome profiling data were at GitHub (https://github.com/ingolia-lab/RiboSeq).<br>Other custom scripts used in this study are available from corresponding author upon reasonable request.

For manuscripts utilizing custom algorithms or software that are central to the research but not yet described in published literature, software must be made available to editors and reviewers. We strongly encourage code deposition in a community repository (e.g. GitHub). See the Nature Research guidelines for submitting code & software for further information.

Data

Policy information about availability of data

All manuscripts must include a data availability statement. This statement should provide the following information, where applicable:

- Accession codes, unique identifiers, or web links for publicly available datasets
- A list of figures that have associated raw data
- A description of any restrictions on data availability

RNA-Seq and ribosome profiling data (GEO: GSE149016) used in this study have been deposited to NCBI. Gene annotation was obtained from Saccharomyces Genome Database (SGD) (https://www.yeastgenome.org).

Field-specific reporting

Please select the one below that is the best fit for your research. If you are not sure, read the appropriate sections before making your selection.

☒ Life sciences ☐ Behavioural & social sciences ☐ Ecological, evolutionary & environmental sciences

For a reference copy of the document with all sections, see nature.com/documents/nr-reporting-summary-flat.pdf

Life sciences study design

All studies must disclose on these points even when the disclosure is negative.

Sample size	No statistical methods were used to predetermine sample size. For RNA-seq and ribosome profiling analyses, two independent biological replicates were analyzed based on standard in the field, which is sufficient for statistical analysis by DEseq to estimate data's dispersion.
Data exclusions	We did not exclude any data for analysis.
Replication	Reproducibility of experiments were confirmed; two replicates in deep sequencing, Western blotting, and Northern blotting data and three independent experiments for the others. Western blotting and Northern blotting data presented in this study are representative results from at least two independent experiments.
Randomization	Randomization is not applicable in this study because there are no static test that requires randomization of samples.
Blinding	Blinding is not applicable in this study because almost all data were acquired by machines or by custom scripts.

Reporting for specific materials, systems and methods

We require information from authors about some types of materials, experimental systems and methods used in many studies. Here, indicate whether each material, system or method listed is relevant to your study. If you are not sure if a list item applies to your research, read the appropriate section before selecting a response.

Materials & experimental systems

n/a	Involved in the study	
☐	☒	Antibodies
☒	☐	Eukaryotic cell lines
☒	☐	Palaeontology and archaeology
☒	☐	Animals and other organisms
☒	☐	Human research participants
☒	☐	Clinical data
☒	☐	Dual use research of concern

Methods

n/a	Involved in the study	
☒	☐	ChIP-seq
☒	☐	Flow cytometry
☒	☐	MRI-based neuroimaging

Antibodies

Antibodies used	Anti-FLAG (Sigma-Aldrich , F3165), anti-Pho8 (Abcam, ab113688), anti-β-actin (Wako, 010-27841), anti-Dpm1 (Invitrogen, A6429), anti-GFP (Roche, 11814460001), anti-Ape1 (Ohsumi lab stock, Hamasaki et al., Cell Struct Funct 2003), anti-digoxigenin-AP (Roche, 11093274910), anti-Gsp1 (ImmuQuest, IQ241), anti-Van1 (a gift from Koji Yoda), and anti-streptavidin-HRP (Abcam, ab7403)
Validation	Anti-FLAG (western blot), anti-Pho8 (western blot), anti-β-actin (western blot), anti-Dpm1 (western blot), anti-GFP (western blot/immunoprecipitation), anti-Gsp1 (western blot), anti-digoxigenin-AP (dot blot/northern blot/western blot etc.), and anti-streptavidin-HRP (dot blot/western blot) were validated by manufacture.<br>Anti-Ape1 is Ohsumi lab stock that was used in previous studies (e.g. Hamasaki et al., Cell Struct Funct 2003; Adachi et al., JBC 2017; May et al., Nat Commun 2020).<br>Anti-Van1 was validated by western blotting in previous papers (Hashimoto and Yoda, BBRC 1997; Yamamoto et al., J Cell Biol 2012).
